# Cell, Isoform, and Environment Factors Shape Gradients and Modulate Chemotaxis

**DOI:** 10.1371/journal.pone.0123450

**Published:** 2015-04-24

**Authors:** S. Laura Chang, Stephen P. Cavnar, Shuichi Takayama, Gary D. Luker, Jennifer J. Linderman

**Affiliations:** 1 Department of Chemical Engineering, University of Michigan, Ann Arbor, Michigan, United States of America; 2 Department of Biomedical Engineering, University of Michigan, Ann Arbor, Michigan, United States of America; 3 Department of Macromolecular Science and Engineering, University of Michigan, Ann Arbor, Michigan, United States of America; 4 Center for Molecular Imaging, Department of Radiology, University of Michigan Medical School, Ann Arbor, Michigan, United States of America; 5 Department of Microbiology and Immunology, University of Michigan Medical School, Ann Arbor, Michigan, United States of America; Washington University, UNITED STATES

## Abstract

Chemokine gradient formation requires multiple processes that include ligand secretion and diffusion, receptor binding and internalization, and immobilization of ligand to surfaces. To understand how these events dynamically shape gradients and influence ensuing cell chemotaxis, we built a multi-scale hybrid agent-based model linking gradient formation, cell responses, and receptor-level information. The CXCL12/CXCR4/CXCR7 signaling axis is highly implicated in metastasis of many cancers. We model CXCL12 gradient formation as it is impacted by CXCR4 and CXCR7, with particular focus on the three most highly expressed isoforms of CXCL12. We trained and validated our model using data from an *in vitro* microfluidic source-sink device. Our simulations demonstrate how isoform differences on the molecular level affect gradient formation and cell responses. We determine that ligand properties specific to CXCL12 isoforms (binding to the migration surface and to CXCR4) significantly impact migration and explain differences in *in vitro* chemotaxis data. We extend our model to analyze CXCL12 gradient formation in a tumor environment and find that short distance, steep gradients characteristic of the CXCL12-γ isoform are effective at driving chemotaxis. We highlight the importance of CXCL12-γ in cancer cell migration: its high effective affinity for both extracellular surface sites and CXCR4 strongly promote CXCR4+ cell migration. CXCL12-γ is also more difficult to inhibit, and we predict that co-inhibition of CXCR4 and CXCR7 is necessary to effectively hinder CXCL12-γ-induced migration. These findings support the growing importance of understanding differences in protein isoforms, and in particular their implications for cancer treatment.

## Introduction

Chemotaxis is a critical physiological and pathological process. Although cells only discern local differences in chemokine concentration via receptor binding, chemoattractant gradients may be maintained over distances much greater than a cell length. These long distance gradients provide a roadmap for leukocytes to reach sites of inflammation or cancer cells to invade and metastasize to distant organs. The simplest notion of chemotactic gradient formation involves secretion of soluble chemokines and diffusion away from their source. *In vivo* gradient formation is fundamentally more complicated and dynamic, involving multiple cell types, chemokine removal by receptors, and interactions with the physical migration “terrain”. To therapeutically target gradient formation and chemotaxis, experimental and computational models are needed to facilitate observation, control, and prediction at all scales: molecular, cellular, and tissue.

Due to the challenge of visualizing and manipulating *in vivo* gradients, many chemotaxis model systems have been developed. Such systems, including Boyden chambers and microfluidic generators, supply stable, defined gradients, but these exogenous, applied gradients may not provide physiologically-relevant gradient formation. To bridge this gap, we recently highlighted an *in vitro* microfluidic source-sink device that exploits *cell-generated* gradients to drive chemotaxis [1, 2] (Figure A in S1 File). Here, we leverage these microfluidic device-based data to develop a computational model and predict which underlying molecular-scale events control *in vivo* gradient generation and ensuing chemotaxis.

The CXCL12/CXCR4 signaling axis is a prime example of how cellular and environmental factors form complex chemoattractant gradients that guide cells to distant locations. This signaling pathway has been implicated as a major driver of metastasis in multiple malignancies [3–8]. In breast cancer, CXCL12 is secreted by both carcinoma-associated fibroblasts and cancer cells in the primary tumor environment and is constitutively expressed in common sites of metastasis, such as bone, lung, and liver [9, 10]. CXCL12 has two known receptors, the G-protein coupled receptor CXCR4 and the atypical chemokine receptor CXCR7 (recently renamed ACKR3). CXCR4 binding to CXCL12 initiates survival, growth, and chemotaxis pathways [11]. Expression of the CXCR4 receptor, which is upregulated on cancer cells in both primary and metastatic tumors, mirrors that of CXCL12, suggesting that CXCR4-bearing cancer cells are actively guided by CXCL12 gradients to exit the primary tumor and metastasize to distant organs [12]. CXCR7 functions in part as a decoy receptor that scavenges and degrades ligand from the extracellular space [13]. CXCR7 is overexpressed on tumor-associated vasculature as well as on subsets of cancer cells in the primary tumor environment, and this receptor has been shown to lower overall CXCL12 levels in tumors [14, 15]. Collectively, these receptor-based interactions between CXCL12, CXCR4, and CXCR7 only partially define the dynamic and complex signaling environment that drives chemotaxis.

Although receptor-based mechanisms of gradient formation and signaling are most well-known, ligand-specific effects may also dictate gradient shape. Much of the current knowledge on CXCL12/CXCR4 signaling focuses on the CXCL12-α isoform, as it is the most prevalent. However, other isoforms have been detected at lower expression levels. Studies of CXCL12 generally have overlooked the existence of six alternatively spliced isoforms (α, β, γ, δ, ε, and φ) that are comprised of an identical N-terminal core and vary by the addition of 1 to 41 largely basic amino acids at unstructured C-termini [16]. Both α and β isoforms have been detected in the primary tumor environment[10], and we recently identified CXCL12-γ in late stage cancer [1]. These isoforms have between 1 and 4 putative basic heparan sulfate binding domains, which affect how well isoforms bind to the migration surface and receptors and may also influence gradient stability and local concentrations [17–20]. Whether or not isoform-specific effects enhance or hinder migration is unclear.

To improve our understanding of how gradients are dynamically shaped and maintained and how cells interact with such gradients, we developed a multi-scale hybrid agent-based model. Multi-scale models have been used to explain emergent properties on the tissue-scale that arise from individual cell decisions informed by the molecular-scale for a wide range of biological phenomena [21–23]. We used *in vitro* data on breast cancer cells migrating in a microfluidic source-sink device to train and validate the model and to predict gradient characteristics. We then used the model to ask: How do active cell properties, such as binding and internalization, shape the gradient? What are the factors that can explain different isoform abilities to elicit chemotaxis? Given what can be discovered in the device, how might these results be used to make predictions for a tumor environment?

Our simulations demonstrate that the decoy receptor CXCR7 dictates gradient magnitude and shape across the device environment due to its strong affinity for CXCL12. Despite a lower effective affinity of CXCL12 for CXCR4, CXCR4-expressing cells shape the local gradient at their leading edge and dynamically change the gradient as they move. In addition to these active-cell factors, simulations identify that properties of strong affinity for the migration surface and a high effective affinity for CXCR4 make the CXCL12-γ isoform a much stronger elicitor of chemotaxis than the α or β isoforms. Our model predicts that CXCR4+ cells move best in short distance, steep gradients that are characteristic of gradients of CXCL12-γ. Furthermore, we predict that blocking CXCR4 and CXCR7 receptors is less effective at inhibiting CXCR4+ migration in gradients of CXCL12-γ compared to the other isoforms. Importantly, these emergent properties still hold true in simulations of a tumor environment, indicating the potential importance of CXCL12-γ in enhancing chemotaxis in cancer.

## Methods

### Cell migration in a microfluidic source-sink device

All *in vitro* experimental data, except those used for model validation, are taken from our previous report of chemotaxis in an *in vitro* microfluidic source-sink device [1]. Briefly, MDA-MB-231 breast cancer cells were individually transduced to express recombinant CXCL12 isoforms (α, β, or γ), CXCR4, or CXCR7. Three cell types, cells secreting one of the CXCL12 isoforms (CXCL12+, source cells), CXCR4-expressing cells (CXCR4+), and CXCR7-expressing (CXCR7+) cells, were patterned in the microfluidic source-sink device. This pattern consisted of one stripe of CXCR4+ cells patterned between one stripe each of CXCL12+ and CXCR7+ cells. Each stripe of cells is 200 μm wide with 200 μm distance between each stripe [1, 2] (Figure A in S1 File). Chemotaxis was quantified by averaging the mean distance CXCR4+ cells moved towards the source cells after 24 hr compared to 0 hr. To manipulate ligand levels, portions of the CXCL12+ cells were replaced with non-secreting cells. These cell dilutions are represented as “Percentage of source cells secreting CXCL12.” Parameters obtained directly from the *in vitro* source-sink device include dimensions of the microfluidic environment, cell density, cell patterning dimensions, and dilution of the number of secreting cells (Table A1 in S1 File).

For this work, we obtained additional data for model validation using the microfluidic source-sink device. For these experiments, we increased gaps between the cell stripes from 200 μm to 400 μm (Figure A in S1 File). Total receptor numbers were quantified using flow cytometry as described in [24] and are shown in Table B in S1 File.

### Multi-scale hybrid agent-based model overview

**Fig 1:**
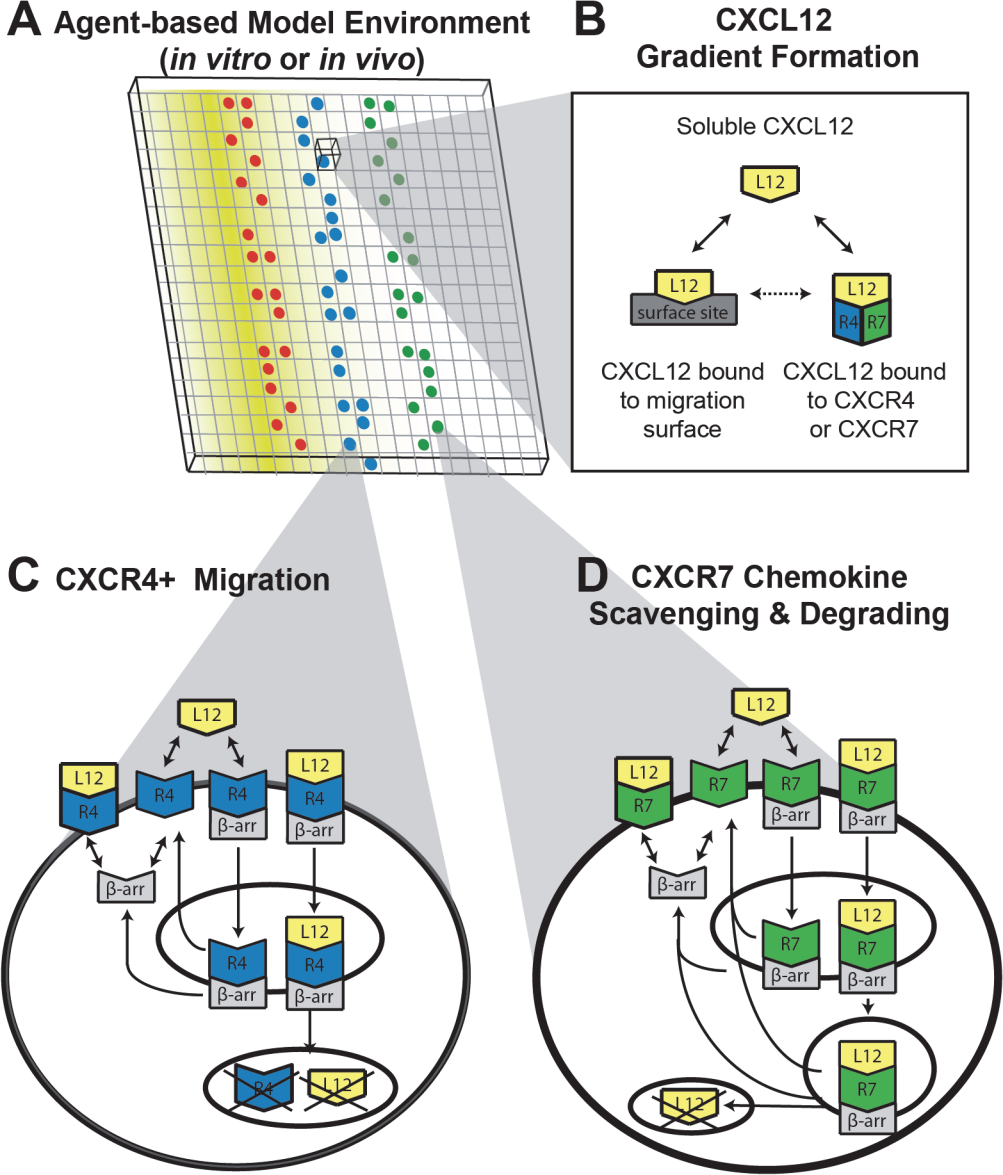
Multi-scale hybrid agent-based model. Model simulates CXCR4+ cell movement in response to CXCL12 gradients. (A) Three types of agents (cells) move in a discrete manner on the surface of a 2D or 3D lattice: CXCL12+, CXCR4+ cells and CXCR7+ cells. CXCL12 gradients are formed and shaped by diffusion and degradation binding to the migration surface (B), and receptor-mediated uptake by CXCR4 (C) and CXCR7 (D). We assume that both soluble CXCL12 and CXCL12 bound to the migration surface are able to bind CXCR4 or CXCR7. Molecule-scale dynamics shown in C and D are described by experiments and model in [32].

We constructed two variants of a multi-scale hybrid agent-based model of CXCR4+ chemotaxis in cell-generated CXCL12 gradients (Fig 1). We represent our microfluidic source-sink device environment (Figure A in S1 File; [1]) with a 3D lattice. CXCL12+, CXCR4+, CXCR7+ cells are represented as discrete agents within the model and move in 2D on the bottom layer of the lattice, the “migration surface” (Fig 1A). We represent the *in vivo* tissue environment with a 2D grid. For both model variants, CXCL12 is secreted into the environment, diffuses and degrades in the extracellular space, and binds to the migration surface and to CXCR4 and CXCR7 receptors. Receptor-mediated uptake of CXCL12 by CXCR4 and CXCR7 further shapes the gradient by removing ligand from the environment. Movement on the cellular-scale is dependent on gradient formation on the tissue-scale and receptor dynamics on the molecular-scale. These three scales are linked by a chemotaxis algorithm that governs CXCR4+ cell movement.

### Model implementation

The model consists of two different layers: an environment layer and an agent layer. The environment is a grid that holds the information for all of the extracellular molecules (soluble and device-bound CXCL12). The agent layer contains the position and molecular-scale information (see Tables A2 in S1 File for all molecular species) for each agent. When an agent interacts with its environment (ie. ligand uptake), the extracellular ligand concentration is the value in the corresponding gridspace of the environment layer.

### Model geometry

The chamber of the experimental microfluidic source-sink device is 20 mm x 2 mm x 0.1 mm (length x width x height) [1] (Figure A in S1 File). In order to model gradient generation and cell movement within the microfluidic source-sink device while minimizing computational cost, we assume symmetry and model a 0.25 mm x 2 mm x 0.1 mm space using a 25 x 200 x 10 grid. The lattice spacing is 10 μm, approximately the size of a single cell, and thus only one cell is allowed to occupy each lattice space at any time. For CXCL12 diffusion, we implement no flux boundary conditions on the four sides that represent the top, bottom, left, and right edges of the device and periodic boundary condition on the front and back edges. Agents reside on the migration surface and move in 2D. For agent movement, we implement no flux boundary conditions on left and right edges, and periodic boundary conditions on the front and back edges. Implementation of these boundary conditions approximate both gradient generation and agent movement in the long microfluidic channel despite the shortened grid (Figure B in S1 File).

For tumor-environment simulations, we use the same lattice framework, but with a 100 x 100 2D grid. We assume that the simulated tumor environment neighbors a similar environment by implementing no flux boundary conditions on all four sides for diffusion and periodic boundary conditions on all sides for agent movement. To assess if gradient predictions in the 2D grid can be extrapolated to those in 3D gradients, we performed some pseudo-tumor simulations with a 100 x 100 x 5 grid, with no flux boundary conditions for diffusion on all sides.

### Secretion and diffusion of soluble CXCL12

We assume a constant and uniform secretion rate per cell (rate constant: *CXCL12sec*); secreted ligands are deposited into the cell-containing compartment. To compare to experimental data, in some simulations we allow only a fraction of CXCL12 cells to secrete CXCL12. The overall secretion rate is the product of the number of secreting cells and the secretion rate constant. Diffusion of soluble CXCL12 (*L*) is described by the diffusion equation.

$$\frac{\partial [L]}{\partial t}=D\left(\frac{\partial^{2}[L]}{\partial x^{2}}+\frac{\partial^{2}[L]}{\partial y^{2}}+\frac{\partial^{2}[L]}{\partial z^{2}}\right)$$ (1)

### Binding of CXCL12 to migration surface

CXCL12 isoforms have different affinities for glycosaminoglycans (γ > β > α), and we assume that the affinity of isoforms to the device surface is in the same order [19, 20]. We use mass action kinetics and assume reversible binding of ligand (*L*) to sites on the migration surface (*S*) to calculate the concentration of ligand bound to the migration surface (*L* ⋅ *S*):

$$\frac{d[L\cdot S]}{dt}=k_{f,L,S}([L][S]-K_{D,L,S}[L\cdot S])$$ (2)

where *k*_*f*,*L*,*S*_ is the forward rate constant for ligand binding to the surface site and *K*_*D*,*L*,*S*_ is the equilibrium dissociation constant for the same (Fig 1B). We assume that ligand cannot bind surface sites that are currently occupied by a cell.

### Extracellular degradation of CXCL12

CXCL12 undergoes protease-mediated degradation in cell media and in extracellular space[25, 26]. For simplicity, we assume that extracellular degradation of soluble and surface-bound CXCL12 occurs with the same first-order kinetics (rate constant *k*_*deg*_):

$$\frac{d[L]}{dt}=-k_{\text{deg}}[L]$$ (3)

$$\frac{d[L\cdot S]}{dt}=-k_{\text{deg}}[L\cdot S]$$ (4)

### Receptor-mediated ligand uptake and dynamics

Receptor internalization and desensitization impacts chemotactic responses as well as gradient shaping, which are both influenced by receptor interactions with β-arrestin [27–31]. To focus on the effects of cell-based gradient shaping, we implement ordinary differential equations (ODEs) based on mass action to describe molecular-scale events for CXCR4+ (Fig 1C) and CXCR7+ cells (Fig 1D). These equations describe ligand binding to receptor, receptor binding to β-arrestin, internalization of β-arrestin-bound complexes, receptor and β-arrestin recycling, and receptor and ligand degradation. The ODEs that describe these events were constructed and validated in previous work ([32] and Tables A2-6 in S1 File). We consider the effective affinity of ligand for receptor to account for binding affinities for the receptor itself and glycosaminoglycans on the cell surface. There are two key differences between CXCR4 and CXCR7 dynamics: (1) β-arrestin binds transiently to CXCR4, whereas it binds tightly to CXCR7, and (2) CXCR4 degradation is ligand-induced, whereas CXCR7 is constitutively recycled (Fig 1C and 1D). Prior to simulation, these CXCR4 and CXCR7 ODEs are solved in the absence of ligand to determine the number of unbound β-arrestin and surface and internalized receptors per cell at steady state, and cells with these quantities are placed on the grid. We did not incorporate other aspects of CXCL12/CXCR4/CXCR7 signaling, such as ligand dimerization and receptor homo- and heterodimers, as there is very limited information on the kinetics of these processes and our previous work showed that they are not required to adequately describe ligand binding and internalization [32]. Dimer formation occurs at a much smaller time scale than ligand-receptor dynamics and dimers are relatively stable over the experimental time frame [33–36], indicating that including kinetics of dimer formation in the model may not be necessary. We did not include CXCR7 signaling, as the migrating cells do not express CXCR7 [2]. Our focus on the model is on the effects of cell-generated gradients, and prior studies also show that chemokine scavenging by CXCR7 on sink cells is sufficient for gradient formation [37–39].

### Random cell movement

Cells move in 2D on the surface of the device or in the tissue environment. At each movement time step, we calculate the next location for each agent. An agent can move to any of the eight lattice spaces in its Moore neighborhood or stay in its current space. To implement random movement for CXCL12+ and CXCR7+ cells, each of the nine lattice spaces is assigned an equal probability for movement. From these individual probabilities, we calculate a cumulative probability distribution that sums to 1. A random number between 0 and 1 is chosen, and the agent moves into the lattice space corresponding to that probability, if the chosen lattice space is empty. Otherwise it remains in its current lattice space. This type of simple random movement algorithm has been employed in numerous models (e.g. [40–43]).

### Chemotaxis algorithm

To replicate experimental *in vitro* data, we needed to capture the motion of hundreds of individual cells over a 24 hr time period in response to a changing chemokine gradient. Specifically, we wanted to understand the role of receptor binding and internalization in that process. For computational tractability we chose a coarse-grained representation where chemotaxis is implemented as a movement probability defined by receptor occupancy and the number of CXCR4 surface receptors.

We build on previous models by assuming that cells sense gradients by comparing differences in receptor occupancy across their length [44–50] and implement this assumption on the discretized environment by calculating the projected receptor occupancy (*RO*) [50, 51] for each lattice space in the Moore neighborhood of a CXCR4+ cell (including its current space):

$$RO=\frac{\left[C\right]}{[C]+K_{D,R4,L12}}*\frac{\left(surfaceCXCR4\right)}{9},$$ (5)

where *[C]* is the sum of soluble and surface-bound ligand concentration in that lattice space [52–55], *K*_*D*,*R4*,*L12*_ is the equilibrium dissociation constant for ligand binding to CXCR4, and *surfaceCXCR4* is the total number of CXCR4 receptors on the cell surface of the agent. We assume a uniform distribution of receptors on the cell surface, and thus 1/9 of the total surface receptors are available to sense concentrations in any of the 9 lattice spaces [56].

To translate differences in receptor occupancy to probability intervals, we first calculate

$$dRO_{i}=\frac{RO_{i}-\text{min}+s}{\sum_{i=1}^{9}RO_{i}-\text{min}+s},$$ (6)

where *RO*_*i*_ is the projected receptor occupancy of the i^th^ lattice space, *min* is the minimum *RO* of the neighborhood and *s* is a chemotaxis sensitivity factor, representing the difference in *RO* to which a cell is sensitive. The sensitivity factor allows cells to respond to absolute differences in receptor occupancy, as opposed to relative differences in receptor occupancy. Similar to the procedure for CXCL12+ and CXCR7+ cells, the *dRO*_*i*_ values are normalized to 1, the cumulative probability distribution is created, and the agent moves into the lattice space corresponding to a random number chosen from 0 and 1 if the chosen lattice space is empty. If *K*_*D*,*R4*,*L12*_ *>>[C]*, or if *K*_*D*,*R4*,*L12*_ *<< [C]* then the calculated receptor occupancy values are similar and cells move essentially randomly.

The inclusion of the chemotaxis sensitivity factor *s* implements the assumption that chemotaxis is dependent on the number of cell surface receptors present at the time of movement [50, 56–58]. We implement *s* as a decreasing linear function of the number of available surface chemokine receptors:

s=m×surfaceCXCR4+b, (7)

where *m* is the slope, *b* is the y-intercept, and *surfaceCXCR4* is as described above. These sensitivity parameters are fit to experimental data. While *s* is not limited to this form, model variants without this simple dependence on receptor number were unable to capture cell migration behavior in device experiments. When the number of available surface chemokine receptors is small, *s* is much greater than the difference in receptor occupancy, and the resulting probability intervals are similar and movement is random. As the number of available surface chemokine receptors increases and *s* approaches zero, chemotaxing cells move based on differences in receptor occupancy alone.

### Initial cell positions

**Fig 2:**
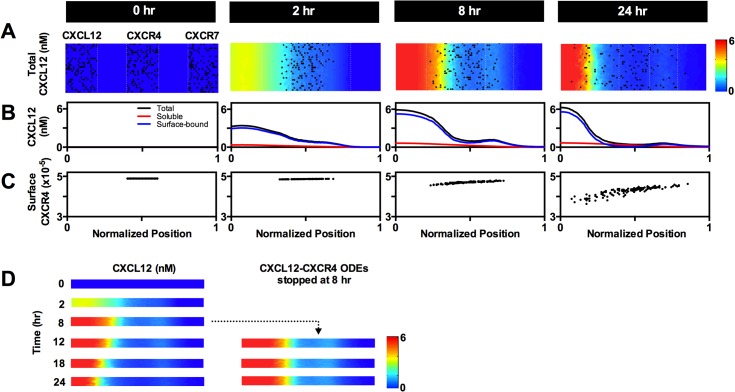
Multi-scale model predicts cell movement, molecular-scale information, and gradient formation within the microfluidic source-sink device. (A) Heat maps depict CXCL12 concentration in the agent layer and are overlaid with agent positions. Initial patterning of CXCL12+, CXCR4+ and CXCR7+ cells is shown at 0 hr. CXCL12+ and CXCR7+ cells, which move randomly, are not shown at later time points for clarity. Concentration values are averaged from 30 simulations. Cell positions shown are from one representative simulation. (B) Contribution of surface-bound (blue) and soluble (red) CXCL12 on total CXCL12 (black) concentration. At 0 hr, all concentrations are 0 nM. Concentration values are averaged from 30 simulations. (C) Number of CXCR4 surface receptors (sum of free, ligand-bound and β-arrestin-bound CXCR4) per individual CXCR4+ cell is plotted at the cell’s position across the width of the device. Data are shown for one representative simulation, the same simulation used for cell positions in (A). (D) Heat maps depicting CXCL12 concentration when CXCL12-CXCR4 binding and internalization ODEs are turned off at 8 hrs. All simulations depicted here were run with parameters from Table A in S1 File. Normalized position of 0 to 1 corresponds to the device width of 0.5mm to 1.5mm.

The simulation grid is initialized with CXCL12+, CXCR4+, and CXCR7+ cells. For device simulations, cells are positioned randomly in stripes, with space in between where no cells are seeded (Figure A in S1 File and Fig 1 and Fig 2A) with a density of 2500 cells/mm^2^, as they are experimentally. For tumor-like simulations, we base cell patterns on histological data from primary breast tumors. Both CXCR4+ cancer cells and CXCL12+ stromal cells typically are in clusters of 4–5 cells [10, 14]. CXCR7+ cells in the tumor environment appear more randomly placed [15]. We determined cell densities by counting the number of CXCL12+, CXCR4+ or CXCR7+ cells in an image frame and divided by the total number of cells within the same frame. In accordance with these images, we initialized tumor-like simulations by randomly placing clusters of CXCL12+ and CXCR4+ cells and individual CXCR7+ cells on the grid. While limited to representative examples of primary breast cancers, these data provide qualitative insights into cell arrangements in human tumors.

### Numerical solution

The overall algorithm of the simulation is depicted in Figure C in S1 File. We use the principle of operator splitting to solve the simultaneous events of diffusion, degradation, and reactions [59–64]. Briefly, this method reduces the diffusion, extracellular binding and degradation, and molecular-scale reaction events to a partial differential equation, linear first order ordinary differential equations, and sets of ordinary differential equations for each lattice space and agent as described above. Within the model workflow, we simulate cell movement using a six minute movement time step over the 24 hr experimental set up, for a total of 240 timesteps. The six minute movement time step was determined by fitting the spread of randomly moving cells to experimental data of CXCR4+ cells moving in the absence of ligand (Figure D in S1 File). Ligand secretion, diffusion, degradation and ligand binding to the migration surface are calculated using 0.1 second timesteps. ODEs depicting receptor-ligand dynamics are solved for each agent using a fourth-order Runge Kutta numerical solver with 0.01 second timesteps. The diffusion and ODE time steps are the maximum time step allowable for solver stability. We use the alternating-direction explicit (ADE) method to solve diffusion as described in previous work [60, 61].

### Uncertainty and sensitivity analysis

We use Latin Hypercube Sampling (LHS) and Partial Rank Correlation Coefficients (PRCC) to identify parameters that have a significant effect on model output. LHS is a stratified non-replacement sampling strategy where all parameters are varied simultaneously [65, 66]. PRCC values are calculated to determine how well the variability of a parameter correlates to a selected model output. PRCC values are between -1 and 1, which are associated with a perfectly negative or perfectly positive correlation between the parameter and model outcome.

To determine how molecular scale events dictate CXCR4+ chemotaxis, we performed sensitivity analyses by varying CXCR4 and CXCR7 kinetic parameters. We used LHS to generate 100 parameter sets, where each parameter was ranged over its known literature values (Tables A3-4 in S1 File). Each set was replicated 10 times to get an average value to perform PRCC analysis.

### Parameter estimation, model fitting and validation

When possible, we use experimental data to determine model parameters. Kinetic and equilibrium parameters for CXCR4 and CXCR7 ODEs are the same as previously published, unless otherwise noted [32] (Tables A3-4 in S1 File). When unavailable, we estimate parameters with values restricted within ranges reported from literature. To determine the isoform-specific effective equilibrium dissociation constant to CXCR4, the equilibrium dissociation constant for binding to the migration surface, secretion rate constant, and *m* and *b* that define the chemotaxis sensitivity factor, we fit the model to experiments measuring CXCR4+ cell migration for each of three CXCL12 isoforms and for several different rates of ligand secretion. The initial estimate for the effective equilibrium dissociation constant to CXCR4 of CXCL12-α was set to the value used in our previously published ODE model; and we assumed that CXCL12 isoforms with higher numbers of heparan sulfate binding domains [17–20] would have higher affinities. The initial estimates for equilibrium dissociation constant for binding to the migration surface of each CXCL12 isoform to the migration surface are based on their reported affinities to heparan sulfate [19]. The initial estimates for isoform-specific secretion rates are based on previously reported experiments using ELISA and bioluminescence-based measurements [1]. We used LHS to generate 100 random parameter sets, ran each parameter set for 20 replications, and chose the parameter set that minimized the squared error between model and experimental data. To validate these isoform-specific fitted parameters, we ran the model for the case where CXCL12+ and CXCR7+ cells are spaced further from the CXCR4+ cells.

### Statistics

All plots and statistical comparisons were created with GraphPad Prism (La Jolla, CA). We plot simulation data as the mean of 30 replications with standard deviation. Additional replications beyond 30 did not significantly change the mean or standard deviation.

## Results

### Multi-scale hybrid agent-based model tracks ligand concentration, cell locations, and molecular-scale information

Using an *in vitro* microfluidic source-sink device dependent on cell-generated gradients to prompt chemotaxis, we previously determined that the strength of CXCR4-mediated chemotaxis is differentially impacted by CXCL12 isoforms and is increased when in the presence of its scavenger receptor, CXCR7 [1, 2]. We hypothesized that this is due to gradient shaping mechanisms that are impacted by isoform-specific secretion rates, ligand binding to surfaces and the rate of ligand scavenging by CXCR7. To quantify these underlying molecular events and how they control gradient shaping and cell responses to chemotaxis, we developed a hybrid agent-based model that links molecular scale interactions to cell population (or tissue) scale outcomes. The model calculates the chemokine concentration gradient over time and position as it is influenced by cell secretion, diffusion and external degradation, receptor-ligand dynamics on moving cells, and binding to the migration surface. Here we present model outputs using the physiological parameters noted in Table A in S1 File.

At the start of simulation, CXCL12+, CXCR4+ and CXCR7+ cells are placed in an environment that lacks ligand (Fig 2A). The model allows us to analyze which molecular-scale events dominate over time. At early times, diffusion and surface binding control the CXCL12 gradient (Figure E, panels A-C in S1 File). At later times, ligand removal by CXCR4 and CXCR7 dynamics are crucial to controlling gradient magnitude and shape; however, they control the gradient at different length scales. CXCR7 is an effective scavenger that binds to CXCL12 with a much greater affinity than CXCR4 [67]. This high affinity for CXCL12 allows CXCR7 to significantly reduce the overall magnitude of CXCL12 concentration in the device and can alter the gradient over distances on the order of millimeters. In contrast, CXCR4 also depletes CXCL12 levels, but primarily in the region where CXCR4+ cells are located (Figure E, panels D-F in S1 File). Sensitivity analysis identified that ligand binding and internalization by CXCR4 and CXCR7 have significant impact on CXCR4+ migration and overall CXCL12 concentration (Table C in S1 File). The contribution of soluble and surface-bound CXCL12 to the total gradient can also be assessed. Most ligand is bound to the migration surface, despite the fact that the affinity of ligand to surface is relatively low (100nM), because there are many surface sites (Fig 2B).

As cells move on the grid, the model dynamically updates molecular scale information. As CXCR4+ cells spend more time in rising CXCL12 levels, ligand binding induces receptor internalization and degradation, resulting in decreasing receptor number over time and position within the device (Fig 2C). Simulations predict that significant losses of surface receptors can occur even at low ligand concentrations. In this representative example, the CXCR4+ cells at the furthermost left and right edges of the device have a difference of ~99,000 surface receptors (a 20% decrease between the rightmost and leftmost cells) at the end of simulation. Much of chemotaxis literature demonstrates chemotaxis in gradients of which cells move, but do not modify, yet we predict that cells actively shape their gradient as they move. As CXCR4+ cells advance towards the source cells, ligand binding, internalization and degradation result in gradient shifting along with the CXCR4+ cells (Fig 2D). Thus, our model captures experimentally observed phenomena and gives mechanistic and molecular insight into chemotaxis. Furthermore, the model offers insight on the differences in contributions of CXCR4 and CXCR7 to the ligand gradient across position and time.

### Migration in cell-generated gradients is dependent on isoform-specific properties

**Fig 3:**
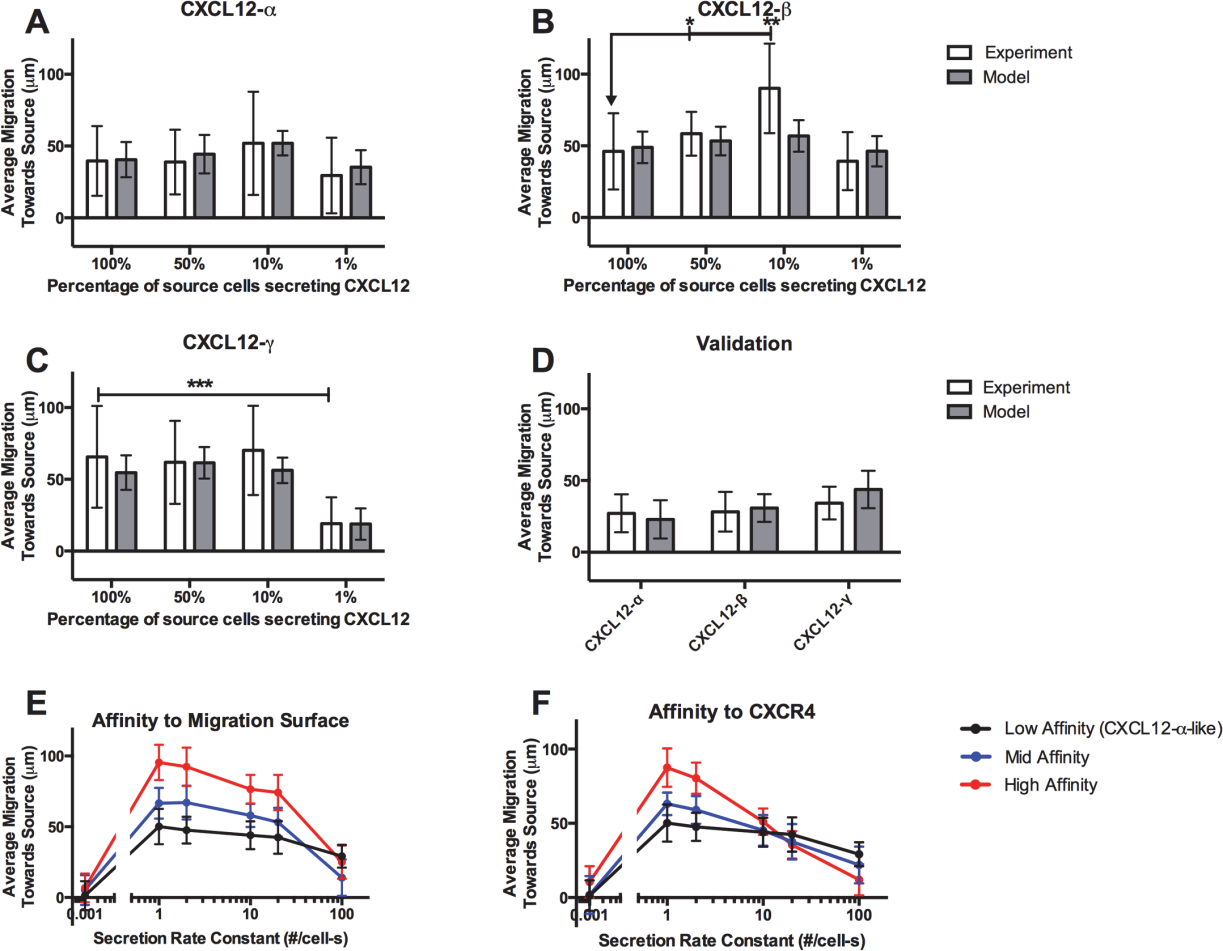
CXCL12 isoform-specific effects on CXCR4+ cell migration. (A-C) Experimental data show isoform-specific differences in average CXCR4+ cell migration as a function of the percentage of source cells secreting CXCL12 in our source-sink device (data from [1]). We fit our model to these data by varying four isoform-specific parameters (chemotaxis sensitivity factor parameter *m*, CXCL12 isoform affinity to the migration surface, CXCL12 isoform effective affinity for CXCR4, and CXCL12 isoform secretion rate). (D) Using the model, we predicted the average migration of CXCR4+ cells within the same source-sink device for when 100% of source cells are secreting and when the distance between the cells stripes is increased from 200 μm to 400 μm. Compared to panels A-C, migration for all isoforms is reduced. We measured the average migration using the source-sink device and find that that it matches model predictions. (E,F) The model predicts CXCR4+ average migration for different secretion rate constants of CXCL12+ cells as (E) ligand affinity to migration surface (Mid affinity *K*_*D*,*L*,*S*_ = 5nM; High affinity *K*_*D*,*L*,*S*_ = 1nM) and (F) ligand affinity to CXCR4 (Mid affinity *K*_*D*,*R4*,*L12*_ = 5nM; High affinity *K*_*D*,*R4*,*L12*_ = 1nM) are increased. The low affinity case for both E and F uses the CXCL12-α parameters listed in Table A in S1 File. Model data are expressed as mean of 30 replications +/- standard deviation. For experiments only: * P < 0.05; ** P < 0.005; *** P < 0.0005.

CXCL12 isoforms have different abilities to generate chemotaxis within the source-sink device. Our experimental data measuring CXCR4+ migration towards the source cells [1] are reproduced in Fig 3A–3C. Notice that with 100% of source cells secreting, gradients of CXCL12-γ can elicit greater migration than gradients of CXCL12-β and CXCL12-α (CXCR4+ migration at 100% secretion, CXCL12-α: 39.6 μm ± 24.3; CXCL12-β: 46.1 μm ± 26.6; CXCL12–γ: 65.7 μm ± 35.5, ordinary one-way ANOVA P-value < 0.0001). CXCL12 isoforms also differ in their migration curves. CXCR4+ migration in gradients of CXCL12-α does not significantly change even when the percentage of secreting cells is decreased by two orders of magnitude, but presents a bell-shaped curve for CXCL12-β and significantly decreases when secretion is diluted to 1% for CXCL12-γ. We hypothesized that four isoform-specific parameters impact migration—secretion rate, affinity for the migration surface, effective affinity for CXCR4, and the chemotaxis sensitivity factor (*s*)—and fit these model isoform-specific parameters to data on CXCR4+ migration for each of the CXCL12 isoforms across the different secretion rates (Fig 3A–3C).

It has been previously noted that CXCL12-γ has a markedly lower secretion rate than the α and β isoforms and a much higher affinity to cell surfaces (due to strong binding to glycosaminoglycans) [1, 19, 20]. As expected, the fit parameters have secretion rates in the order of α>β>>γ and affinities to the migration surface of γ>>β>α (Table 1). Initially, we refrained from varying the effective ligand affinity for CXCR4 and the chemotaxis sensitivity factor parameters, as differences among isoforms are not well established. However, a reasonable fit for CXCL12-γ data was not possible without increasing the effective affinity of CXCL12-γ to CXCR4 as well as slightly decreasing the slope (*m*) of the chemotaxis sensitivity factor (resulting in more sensitive migration) for the CXCL12–γ isoform. Although measurements of CXCL12 isoform binding determined that the affinity of CXCL12-γ to CXCR4 is lower than the other isoforms [20], it has been suggested that the high affinity by which CXCL12–γ binds to cell-surface heparan sulfates may confer a higher effective affinity to CXCR4 than the other isoforms [17]. Limited data show differences in signaling between isoforms, and indicate that binding of the γ isoform to CXCR4 elicits more β-arrestin signaling than the other isoforms [1, 17, 68]. The fitted values for ligand affinity to the migration surface and secretion rate constants between isoforms are in accordance with experimental data [1, 17, 19].

**Table 1:** Isoform-specific fitted parameters.

Parameter	Definition	CXCL12-α	CXCL12-β	CXCL12-γ
K_D,L,S_ (nM)	Equilibrium dissociation constant for surface site binding	100	20	5
CXCL12sec (#/cell-s)	Secretion rate constant	20	15	5
K_D,R4,L12_ (nM)	Effective equilibrium dissociation constant for CXCR4 binding	40	40	10
m	Chemotaxis sensitivity factor slope	-6.73x10^-3^	-6.73x10^-3^	-6.25x10^-3^
b	Chemotaxis sensitivity factor y-intercept	3.5x10^3^	3.5x10^3^	3.5x10^3^

Next, we predicted the effect of increasing the distance between the initial cell positions for all three CXCL12 isoforms on CXCR4+ migration (Fig 3D). Compared to migration using 200 μm spacing, CXCR4+ migration using 400 μm spacing is reduced for all isoforms. We then used the *in vitro* source-sink device under these conditions to measure the average CXCR4+ migration and found that the model predictions agree well with experimental data. Thus, our model accurately describes migration in our microfluidic source-sink device based on isoform-specific differences, placement of cells, and a broad range of CXCL12 secretion. In addition, the fitted parameters support that isoforms differ in their binding to the surface, binding to CXCR4, secretion rates, and sensitivity to movement, with the CXCL12–γ in particular displaying a much higher affinity to the migration surface and effective affinity to CXCR4 compared to the other isoforms.

### Non-specific and receptor binding are both critical to migration

Because our computational model suggested that isoforms differ in binding to the migration surface and to CXCR4, we varied these two parameters to explore how ligand properties affect the chemokine gradient and subsequent chemotaxis. Increasing ligand affinity to the migration surface (decreasing *K*_*D*,*L*,*S*_) creates steeper gradients and results in an upward shift of the chemotaxis curve (Fig 3E). Increased affinity to the migration surface can explain why CXCR4+ cells move furthest in gradients of CXCL12-γ, followed by—β, then—α at 100% secretion. Examination of the CXCL12 gradients reveals that despite lower secretion rates, gradients of CXCL12-β and—γ have higher overall concentrations and steeper gradients due to enhanced binding to the migration surface (Figure F in S1 File). Increasing the ligand effective affinity to CXCR4 results in an upward shift of the chemotaxis curve (Fig 3F). As ligand effective affinity to CXCR4 increases (decreasing *K*_*D*,*R4*,*L12*_), cells chemotax more efficiently in lower concentrations. However, at higher concentrations, surface receptors are downregulated, reducing migration. Together with secretion rate, effective affinity for CXCR4 can explain the isoform-specific chemotaxis curve shapes. CXCL12-α has a low effective affinity to CXCR4 and mid-level secretion rate constant (20 molecules/cell-s) resulting in a relatively flat chemotaxis curve. We could not reproduce the steep bell-shaped curve for CXCL12-β, but note the large error bars for the 10% condition. (A lower secretion rate combined with a high affinity to CXCR4—outside current literature ranges—would achieve a bell-shaped curve.) Finally, CXCL12-γ is secreted at a low rate and has a high effective affinity for CXCR4. The significant decrease at 1% secretion compared to 100% is due to its high effective affinity for CXCR4 resulting in maximal chemotaxis of the CXCL12-γ at 100%, and then a drop off at 1% due to low levels of ligand. These findings confirm that isoform-specific differences in binding to both the migration surface and to CXCR4 significantly control CXCR4+ migration.

### Migration is sensitive to the number of CXCR4 receptors, and less sensitive to CXCR7

**Fig 4:**
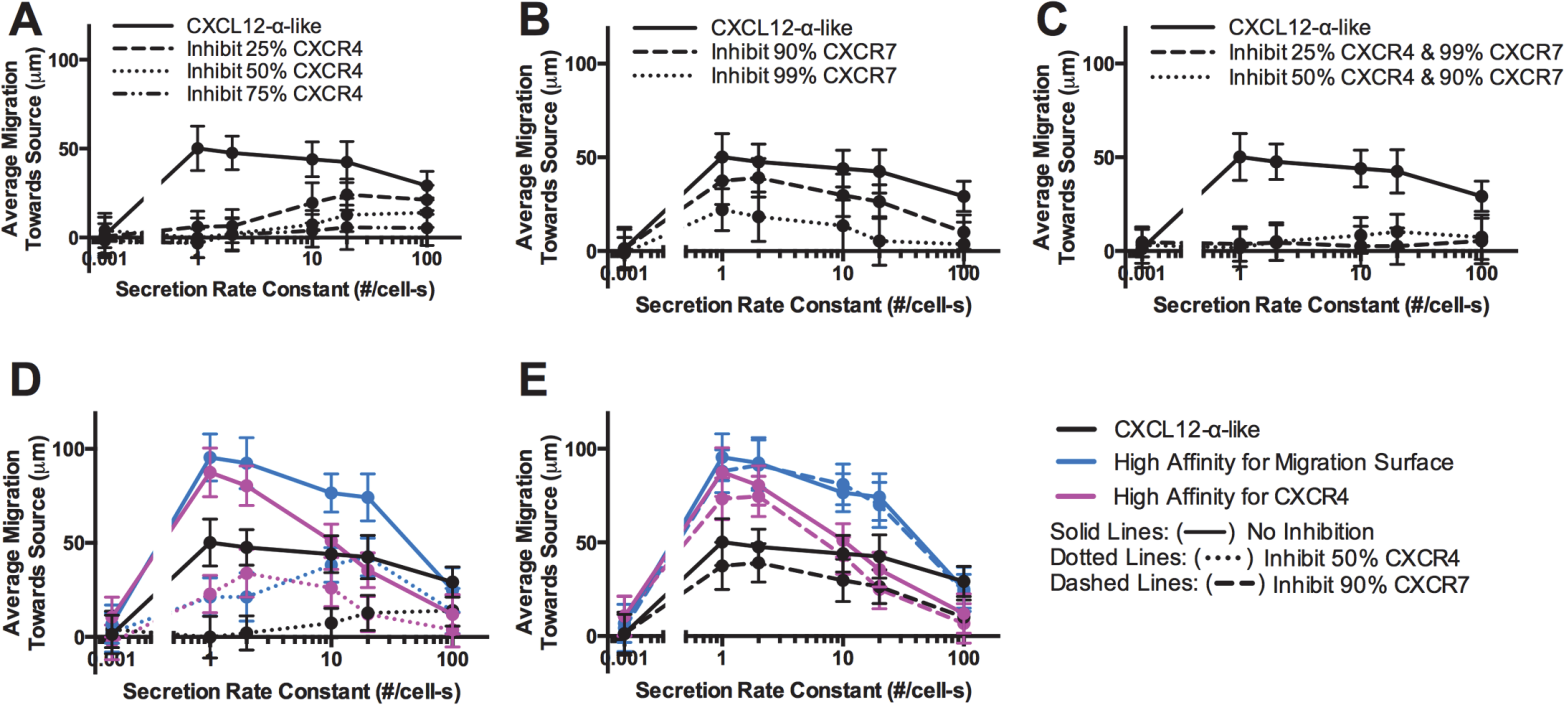
Blocking migration to high affinity ligands is more difficult than to low affinity ligands. Average migration of CXCR4+ cells for different secretion rate constants of CXCL12-secreting cells as (A) CXCR4, (B) CXCR7 or (C) both CXCR4 and CXCR7 are blocked. Blocking 50% of CXCR4 receptors (D) or 90% of CXCR7 receptors (E) is not as effective at reducing CXCR4+ migration when the ligand has a high affinity for CXCR4 (*K*_*D*,*R4*,*L12*_ = 1nM) and when the ligand has a high affinity for the migration surface (*K*_*D*,*L*,*S*_ = 1nM). Data are expressed as mean of 30 replications +/- standard deviation.

Potential therapeutic strategies for reducing chemotaxis include blocking CXCR4 or CXCR7 [57, 69]. Using CXCL12-α-like parameters listed in Table A in S1 File (and determined above), we find that migration is sensitive to the number of CXCR4 and less so to the number of CXCR7 receptors. Blocking as few as 25% of CXCR4 receptors is sufficient to completely inhibit migration at low secretion rates, but is less effective at inhibiting migration at higher secretion rates (Fig 4A). Stronger inhibition of migration across all secretion rates requires blocking more than 50% of CXCR4 receptors. In contrast, nearly all CXCR7 receptors (90–99%) must be blocked to effectively reduce CXCR4+ cell migration at high secretion rates (Fig 4B). Note that due to the high starting numbers of receptors in the transfected cells modeled in the simulation, 50% blocking of CXCR4 and 90% blocking of CXCR7 correspond to ~10^5^ available receptors per cell (3.55x10^5^ and 2x10^5^ receptors per cell for CXCR4 and CXCR7, respectively). Cancer cells have on the order of 10^3^–10^5^ receptors per cell [70, 71]. Clearly strong inhibition of migration is difficult with receptor numbers on the higher end of the range, but may require a smaller percentage of receptors to be blocked to effectively reduce migration on the lower end of the range. Blocking CXCR7 has a greater effect on CXCR4+ migration at high secretion rates and a smaller effect on CXCR4+ migration at lower secretion rates, which is consistent with our *in vitro* data using CXCR7+ cells unable to internalize CXCL12-CXCR7 complexes [1]. At lower secretion rates, the gradient is primarily controlled by diffusion and ligand binding to the migration surface and CXCR7 has little effect. At high secretion rates, CXCR7 plays a significant role in diminishing the overall concentration in the device and limiting CXCR4 internalization (Figure G in S1 File). By blocking both CXCR4 and CXCR7, migration can be inhibited across all secretion rates (Fig 4C), suggesting that a combination of CXCR4 and CXCR7 blocking may work as a treatment strategy to effectively hinder migration.

### Inhibition of CXCR4+ migration is isoform-specific

Next, we examined if inhibition of CXCR4+ cell migration by blocking CXCR4 or CXCR7 is isoform-specific. We already established that high ligand affinity to the migration surface or to CXCR4, which is characteristic of the γ isoform, elicits higher CXCR4+ cell migration (Fig 3). Blocking 50% of CXCR4 receptors is effective at reducing migration across secretion rates for ligand with higher affinity to the migration surface as well as higher affinity to CXCR4 (Fig 4D), but not as well as with lower affinities to the surface or to CXCR4 (CXCL12-α-like). Furthermore, model simulations blocking CXCR7 with isoforms having elevated affinities to the migration surface and to CXCR4 reveal that the role of CXCR7 in modulating migration may also be isoform-specific (Fig 4E). Although blocking 90% of CXCR7 markedly decreases migration for the CXCL12-α-like parameters, it has much less of an effect in modulating migration for ligand with higher affinities to the migration surface and to CXCR4, further highlighting the potential importance of understanding the role of high affinity ligands like CXCL12-γ within the tumor environment. Two questions arise from these *in vitro* and *in silico* studies: do isoform gradients form in the tumor environment and what is the effect on CXCR4+ cell migration?

### Gradients produced by tumor-like cell arrangements

**Fig 5:**
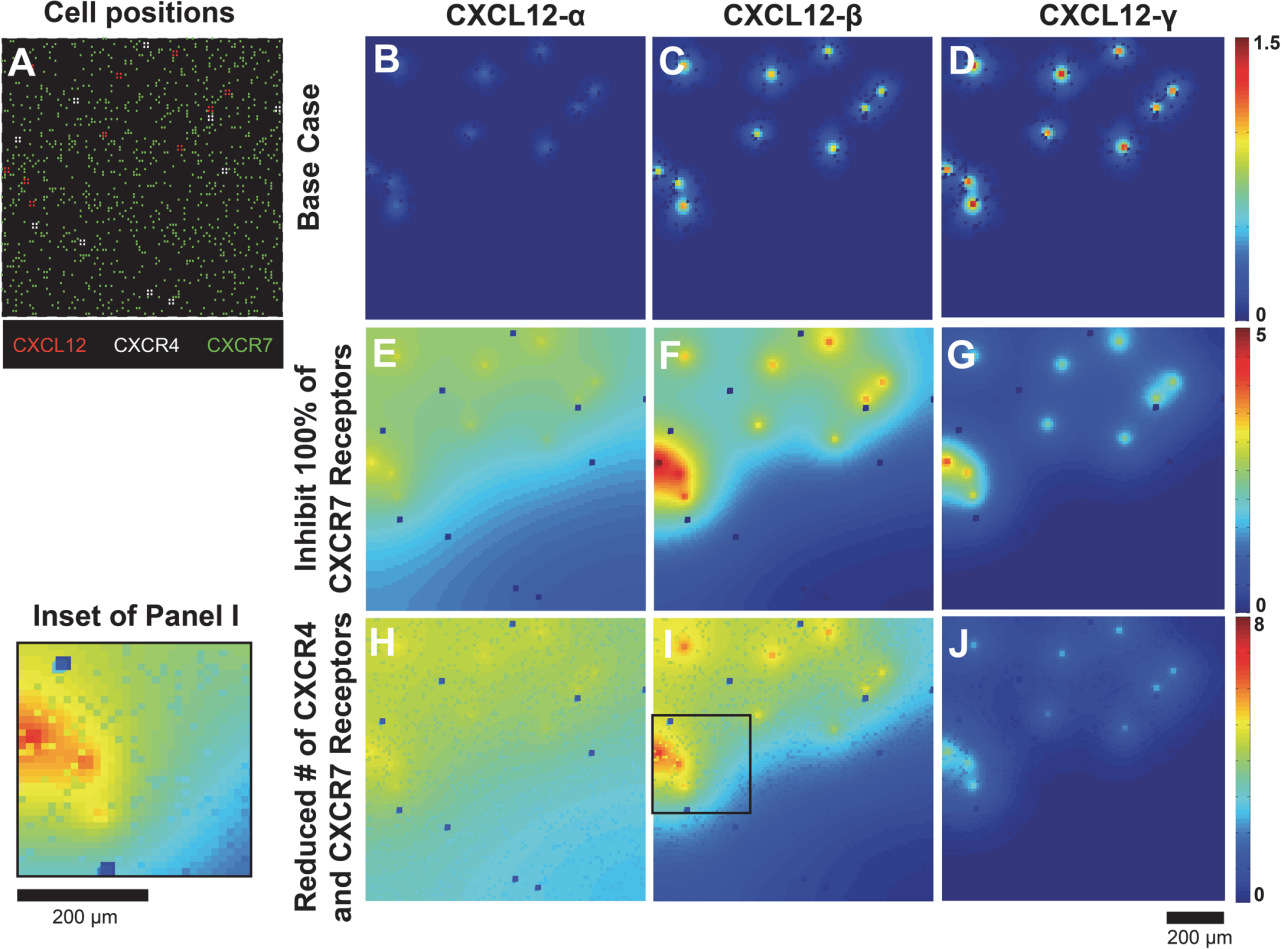
Gradients form even in disorganized cell structures representative of tumors. (A) Positions of CXCL12+ (red), CXCR4+ (white) and CXCR7+ (green) cells in simulation, based on histological data from literature [10, 14, 15]. We predicted chemokine gradients using same parameters as used in the device (Table A in S1 File) for CXCL12-α (B), CXCL12-β (C) and CXCL12-γ (D). Gradients when CXCR7+ cells are replaced with non-receptor bearing cells for CXCL12-α (E), CXCL12-β (F) and CXCL12-γ (G). Gradients using a reduced number of CXCR4 and CXCR7 receptors (5x10^3^ per cell) for CXCL12-α (H), CXCL12-β (I) and CXCL12-γ (J). Gradients shown are at 24 hr; cells were immobile throughout the entire time period. Data are expressed as mean concentration using 30 replications.

We have focused up to this point on cell migration observed in our microfluidic source-sink device, where cells are initially arranged in stripes. We next wanted to explore the implications of our findings for gradient formation and cell migration in a more tumor-like environment. To do this, we patterned cells to mimic the disorganization of CXCL12+, CXCR4+, and CXCR7+ cells observed in invasive breast cancer. We arranged CXCL12+, CXCR4+ and CXCR7+ cells on the simulation grid based on histological data (Fig 5A) [10, 14, 15]. For our initial set of simulations, all cells were immobile and we focused on generation of chemokine gradients within a 24 hr time period.

We first examine gradients using the same parameter values and receptor numbers as in the device (Table A in S1 File). Predicted gradients at 24 hr are shown in Fig 5B–5D for the three isoforms. Similarly to the microfluidic ource-sink device (Figure F in S1 File), gradients of CXCL12-β (Fig 5C) and-γ (Fig 5D) are steeper and have higher maximum concentrations than gradients of CXCL12-α. When CXCR7 is removed from the grid (Fig 5E–5G), gradients for all isoforms become more diffuse, but have the most change for CXCL12-α and CXCL12-β. As CXCR7 has the most influence in gradient shaping when the ligand affinity for the extracellular space is low and when secretion rates are high, CXCR7 is more necessary to shape gradients of CXCL12-α and CXCL12-β than that of CXCL12-γ.

The simulations in Fig 5B–5G used receptor numbers characteristic of the transfected cell lines used in our microfluidic source-sink device experiments (CXCR4, 7.1x10^5^; CXCR7, 2x10^6^). Next, we ran simulations to predict gradients for lower receptor levels (5x10^3^ receptors/cell) that represent the lower range of receptor overexpression in cancer (Fig 5H–5J) [70, 71]. Gradients predicted for receptor numbers in this pathological range are similar to gradients predicted for environments that lack CXCR7. We note that CXCR4+ and CXCR7+ cells are able to produce microgradients, as they internalize ligand in the lattice space where they are placed (highlighted by the Inset of Panel I). These model predictions show that qualitative differences between gradients of CXCL12 isoforms determined in the microfluidic source-sink device hold true in the more disorganized cell arrangements found in tumors, and that secretion rates and ligand binding to the migration surface controls gradient shape and scope.

### Steep short-distance gradients provide better homing than shallow long-distance gradients

**Fig 6:**
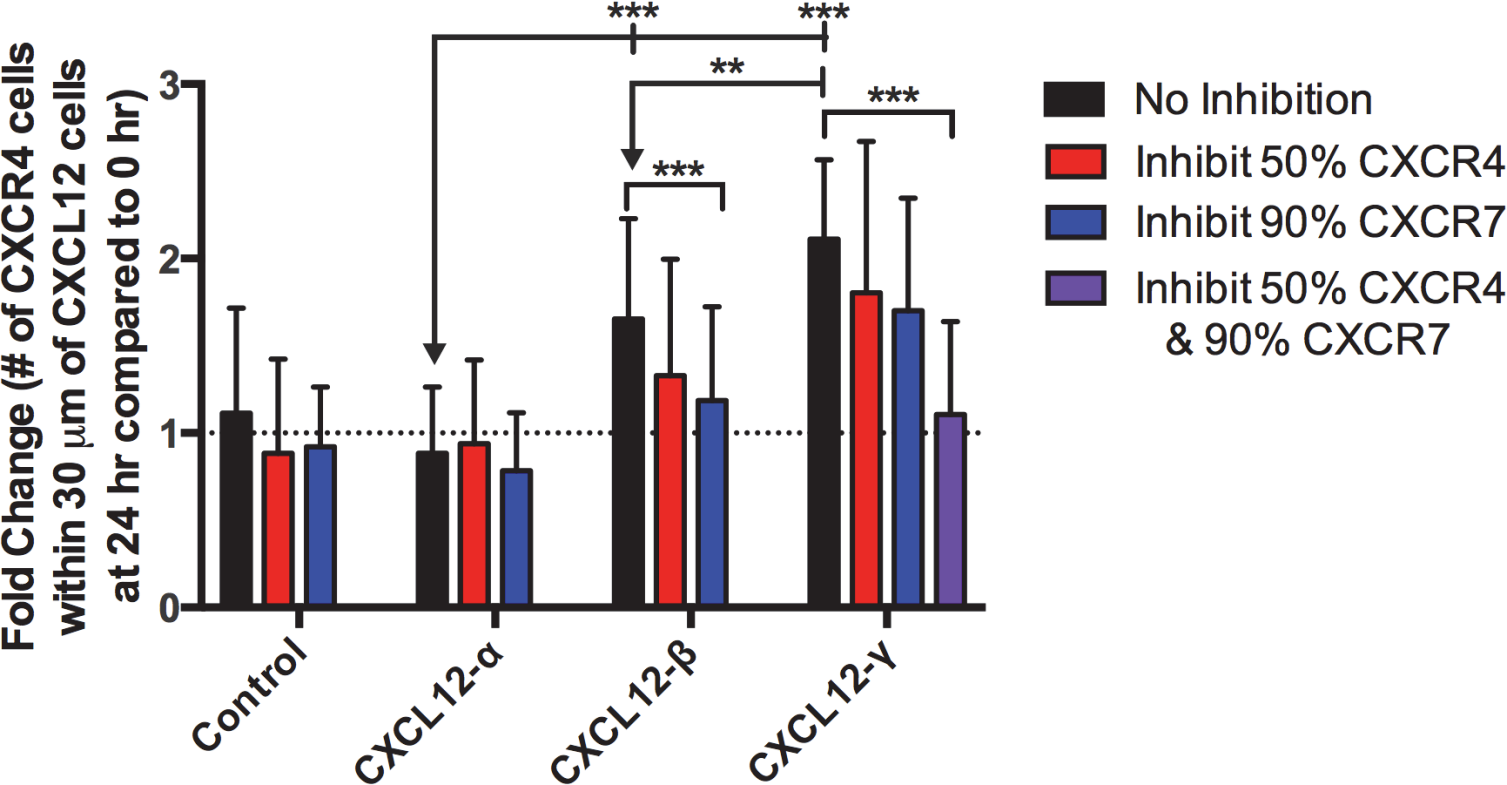
CXCL12-γ enhances migration in tumor-like simulations. Model predictions of directed CXCR4+ migration in a tumor-like environment. Number of CXCR4+ cells within 30 μm of a CXCL12 cluster at 24 hr represented as fold change (in comparison to number at 0 hr). Each simulation had a randomly generated pattern. CXCL12 and CXCR7+ cells remained static throughout the entire time period, whereas CXCR4+ cells were allowed to move. All parameters are the same as those used for gradient predictions in Fig 5H–5J, with reduced numbers of CXCR4 and CXCR7 (5x10^3^ per cell), except that the chemotaxis sensitivity factors (for all isoforms: *m* = -6.85x10^-3^, *b* = 25) were altered to increase sensitivity to migration. Control simulation lacks CXCL12 on the grid. Data are expressed as mean of 30 replications +/- standard error of the mean. Arrows represent statistics using Student’s T-test, brackets represent statistics using ANOVA. * P < 0.05, ** P < 0.0005, *** P < 0.0005.

Continuing with the parameters relevant to receptor overexpression in cancer (as in Fig 5H–5J), we allowed CXCR4+ cells to move (Fig 6). We quantified chemotaxis by normalizing the number of CXCR4+ cells within 30 μm of a CXCL12 cluster at 24 hr to the number at the start of simulation. Similarly to the microfluidic source-sink device, we find that CXCR4+ cells move best in gradients of CXCL12-γ, followed by CXCL12-β, and CXCL12-α. The shallow long-range gradients of CXCL12-α do not promote migration in this cell arrangement and for these low receptor numbers; however, using a higher number of total receptors results in increased migration of CXCR4+ cells in CXCL12-α gradients (Figure H in S1 File), indicating that gradients formed with CXCL12-α-like properties can induce chemotaxis when combined with increased receptor signaling. We also explored which isoform-specific parameters are most responsible for increased migration in gradients of CXCL12-β and—γ by systematically replacing one of the ligand-related parameters of CXCL12-α for that of γ (Figure H in S1 File). Similarly to the device simulations, we found that increased effective affinity for CXCR4 and for the migration surface promotes CXCR4+ chemotaxis towards CXCL12 producing cells. Higher migration in gradients of CXCL12-γ and-β also mirror higher levels of surface CXCR4. The average number of CXCR4 surface receptors per cell is 10 times higher in gradients of CXCL12-γ than in CXCL12-α (850 compared to 86 CXCR4 surface receptors per cell).

Inhibition of either CXCR4 or CXCR7 results in a significant decrease in CXCR4+ migration in gradients of CXCL12-β. Given that single inhibition of CXCR4 or CXCR7 was relatively ineffective at limiting chemotaxis in CXCL12-γ gradients, we performed co-inhibition of CXCR4 and CXCR7, resulting in complete suppression of CXCR4+ movement (P-value = 0.52 at 24 hr compared to at 0 hr). To understand how these results on the 2D grid apply to the 3D environment, we performed additional simulations with the same parameters (as in Fig 5H–5J), but with a 3D dimension for diffusion, using a grid size of 100x100x5 instead of 100x100x1. The gradient trends remain the same: CXCL12-γ and-β promote shorter and steeper gradients than CXCL12-α. When CXCR4+ cells within this expanded grid were allowed to move, we found that the 2D migration trends hold true in 3D (Figure I in S1 File). Taken together, these results demonstrate that CXCL12 isoforms form gradients that differ in shape, magnitude and scope even within the disorganized pattern of cancer cells present in some tumors. Moreover, we call attention to the importance of CXCL12-γ within the tumor environment, as its high ligand binding properties elicits higher CXCR4+ migration and requires interventions such as co-inhibition of CXCR4 and CXCR7 to significantly reduce migration.

## Discussion

Chemotaxis plays a critical role at major stages of cancer progression. Angiogenesis required to supply the tumor with nutrients is directed by external gradients of cytokines such as vascular endothelial growth factor [72–75]. Tumor-promoting macrophages are recruited from blood to tumor sites by CCL2 and CCL5 [76]. Expression of chemokines and their receptors, such as CXCL12/CXCR4 and CCL21/CCR7, in both primary and metastatic tumors suggest that chemokine receptor-bearing cells actively enter the circulation and home to metastatic sites [12]. While it is well established that cells move in a directed manner towards chemoattractants, little is known about the shape and magnitude of chemoattractant gradients in tissue. Understanding gradient dynamics and cell responses to these gradients is imperative to targeting cancer metastasis.

Our multi-scale hybrid agent-based model calculates gradient formation as it is shaped by molecular-scale events and cellular behavior. We trained and validated the model on an *in vitro* microfluidic source-sink device that capitalizes on gradients formed by actively secreting CXCL12+ cells, migrating CXCR4+ cells, and scavenging CXCR7+ cells. We used the model to gain insight on why CXCL12 isoforms have differential effects on migration. Recent data suggest that different isoforms of CXCL12 may have distinct outcomes for cancer [1, 20, 77]. Our simulations demonstrate that ligand affinity for the migration surface and for CXCR4 significantly impact migration. Gradients of CXCL12-γ prompt greater migration of CXCR4+ cells than CXCL12-α in both the microfluidic source-sink device and the pseudo-tumor simulations and can in part be explained by high affinity for both the migration surface and receptor. High affinity for the surface results in steeper gradients for cells to navigate, and the high effective affinity for the receptor results in a shift of the chemotaxis curve that allows higher migration even at lower secretion rates. In addition, cells in gradients of CXCL12-γ maintain higher levels of cell surface CXCR4 than when in gradients of the other CXCL12 isoforms. This is analogous to reports of enhanced chemotaxis with higher cell surface CXCR4 in the pathological setting of WHIM syndrome (warts, hypogammaglobulinemia, infections, and myelokathexis). The cytoplasmic tail of CXCR4 is truncated in WHIM syndrome, resulting in reduced internalization,higher number of CXCR4 surface receptors, and enhanced chemotaxis of leukocytes in response to CXCL12 [3, 78]. These factors may explain why CXCL12-γ is better at eliciting chemotaxis of immune cells and endothelial progenitors compared to other isoforms [20, 79].

The properties that make CXCL12-γ better at eliciting chemotaxis also make it harder to inhibit. Blocking CXCR4 or CXCR7 is less effective at inhibiting CXCR4+ migration when the ligand (like CXCL12-γ) has a high affinity for the migration surface or for CXCR4. Our pseudo-tumor simulations demonstrate that blocking both CXCR4 and CXCR7 receptors may be a potential method to inhibit migration in such gradients. In addition to having a higher affinity for the migration surface and CXCR4, CXCL12-γ is present at lower levels within the tumor environment [77], and lower levels of CXCL12 correlate with increased metastasis in mouse models and worse prognosis in breast cancer [14, 15, 77, 80, 81]. The combined effects of low expression and high binding characteristics make CXCL12-γ a worthwhile target for study in cancer metastasis.

More broadly, our simulations highlight the importance of non-specific ligand binding to extracellular space. We predict that the majority of the chemokine presented to cells is surface-immobilized, even for ligands with ostensibly lower affinities to surface-sites as CXCL12-α. These findings are consistent with *in vivo* findings of stable and functional haptotactic gradients [82]. Many extracellular matrix proteins, such as heparan sulfate and collagens, have been shown to be elevated in cancer [83], but much of the research focus is on their mechanical properties. Our simulations indicate that the high presence of extracellular matrix proteins in cancer may support the steep, short-distance gradients that more efficiently regulate chemotaxis.

One surprising result is that cells shape their own gradient as they move. Largely due to the difficulty of visualizing gradients, most reports of CXCR4+ chemotaxis overlook the participation of CXCR4-bearing cells in shaping their own gradient. Here we use our agent-based model to observe and confirm that cell clusters of CXCR4 actively shape their own gradients. In the *in vitro* microfluidic source-sink device, the collective migrating cells maintain gradient steepness locally at the leading edge. In the pseudo-tumor simulations, we find that individual CXCR4+ cells deplete ligand in their local environment. There is currently little literature on the potential for cells to shape their own gradient [38, 84]; this may be one mechanism by which migrating cells can promote migration of follower cells [85].

While the receptor-ligand binding and internalization dynamics were constructed and validated on quantitative experimental data [32], we recognize that the model uses a simplified view of the complex interactions among CXCL12, CXCR4 and CXCR7. All of these proteins exist in both monomer and dimer forms that may have further implications on the strength of chemotaxis response or switches to other biological responses [33–35, 86, 87]. The presence of heparan sulfate, which was incorporated in the model with device surface-sites as well as the increased effective affinity of CXCL12 to CXCR4, further complicates the picture by inducing CXCL12 dimers and may alter CXCR4 recognition of the ligand [88, 89]. As we gain more knowledge as to how these complicated interactions affect cell responses, we will be able to model more complicated behaviors of migrating cells within CXCL12 gradients.

Ultimately, we are interested in what gradients may look like in the tumor environment. Gradients form even with disorganized tissue architecture present in cancers. We find that short-distance, steep gradients promote greater chemotaxis towards chemokine-secreting cells than shallow gradients maintained over longer distances. Only a few reports have successfully visualized *in vivo* gradients [82, 90, 91], but characteristics such as gradient shape and magnitude have not been quantified. As imaging techniques such as intravital microscopy improve, we should be able to gain more information on gradient formation over time and position in living tissues. To improve our understanding of the factors that modulate chemotaxis in tumors, it will be important to gather more histological data on the numbers and patterning of secreting and receptor-bearing cells in the tumor environment. Improvements to the model should capture matrix interactions in a packed cell environment, the mechanics of cell movement in a dense tissue environment, possible gradients of glycosaminoglycans, and isoform-specific protection from degradation. The integration of computational and experimental work to understand *in vivo* gradient formation will also provide insights to allow modulation of gradients for therapeutic purposes.

## Supporting Information

S1 File Supporting Information. (PDF)
