# Correction: Cell, Isoform, and Environment Factors Shape Gradients and Modulate Chemotaxis

**DOI:** 10.1371/journal.pone.0174189

**Published:** 2017-03-14

**Authors:** S. Laura Chang, Stephen P. Cavnar, Kathryn E. Luker, Shuichi Takayama, Gary D. Luker, Jennifer J. Linderman

Kathryn E. Luker should be included in the author byline. She should be listed as the third author and her affiliation is: Department of Radiology, University of Michigan Medical School, Ann Arbor, Michigan.

The correct citation is: Chang SL, Cavnar SP, Luker KE, Takayama S, Luker GD, Linderman JJ (2015) Cell, Isoform, and Environment Factors Shape Gradients and Modulate Chemotaxis. PLoS ONE 10(4): e0123450. doi:10.1371/journal.pone.0123450

In the Author Contributions section, Kathryn E. Luker (KEL) should be listed as one of the persons who conceived and designed the experiments and who analyzed the data.
