# Supplementary material for: Cell, Isoform, and Environment Factors Shape Gradients and Modulate Chemotaxis
Source: PLoS One. 2015 Apr 24;10(4):e0123450. doi: 10.1371/journal.pone.0123450 (PMC4409393; doi:10.1371/journal.pone.0123450)
Supplement: S1 File — (PDF) [file pone.0123450.s001.pdf]

Cell, isoform, and environment factors shape gradients and modulate chemotaxis

S. Laura Chang, Stephen P. Cavnar, Shuichi Takayama, Gary D. Luker, Jennifer J. Linderman

S1 File. Supporting Information

Figure A in S1 File. Microfluidic source-sink device
Figure B in S1 File. Agent-based model boundary conditions and validation for device setup
Figure C in S1 File. Model workflow
Figure D in S1 File. Validation of movement time step
Figure E in S1 File. Contribution of gradient-shaping events
Figure F in S1 File. Comparison of gradients within device between CXCL12-α, -β, and -γ
Figure G in S1 File. Effect of CXCR7 on CXCR4 surface receptors
Figure H in S1 File. CXCR4+ movement in gradients of CXCL12-α tumor simulations with adjusted parameters
Figure I in S1 File. Tumor simulations using an extended grid
Table A in S1 File. Multi-scale agent-based model parameters
- **Table A1 in S1 File.** Multi-scale model parameters
- **Table A2 in S1 File.** Molecular species involved in ligand-binding and internalization dynamics of CXCR4 and CXCR7
- **Table A3 in S1 File.** Description and values of CXCR4 kinetic and equilibrium parameters
- **Table A4 in S1 File.** Description and values of CXCR7 kinetic and equilibrium parameters.
- **Table A5 in S1 File.** Ordinary differential equations describing events of ligand binding and receptor dynamics
- **Table A6 in S1 File.** Ordinary Differential Equations describing the change in the concentration of species in CXCR4+ and CXCR7+ cells over time

Table B in S1 File. Total receptor numbers for the MDA-MB-231 cells transfected to express CXCR4 and CXCR7 used in the device as determined by flow cytometry
Table C in S1 File. Sensitivity Analysis for CXCR4 and CXCR7 molecular parameters

Figure A in S1 File. Microfluidic source-sink device

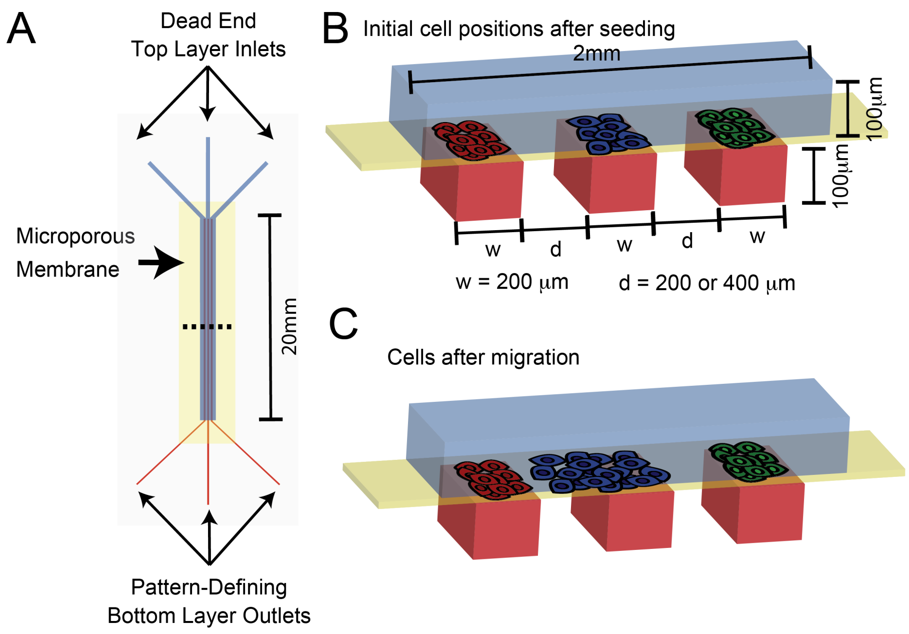

(A) Bird's eye view of *in vitro* microfluidic source-sink device. Cells are patterned into stripes of 200 µm width by use of inlet and outlet ports as described in [1,2]. (B) View of CXCL12+ (red), CXCR4+ (blue), and CXCR7+ (green) cells in device after initial seeding. The spacing between the cell stripes can be 200 µm (used for fitting experiments) or 400 µm (used for validation experiments). (C) Over time, CXCR4+ cells move in the direction of the CXCL12+ cells.

Figure B in S1 File. Agent-based model boundary conditions and validation for source-sink device setup

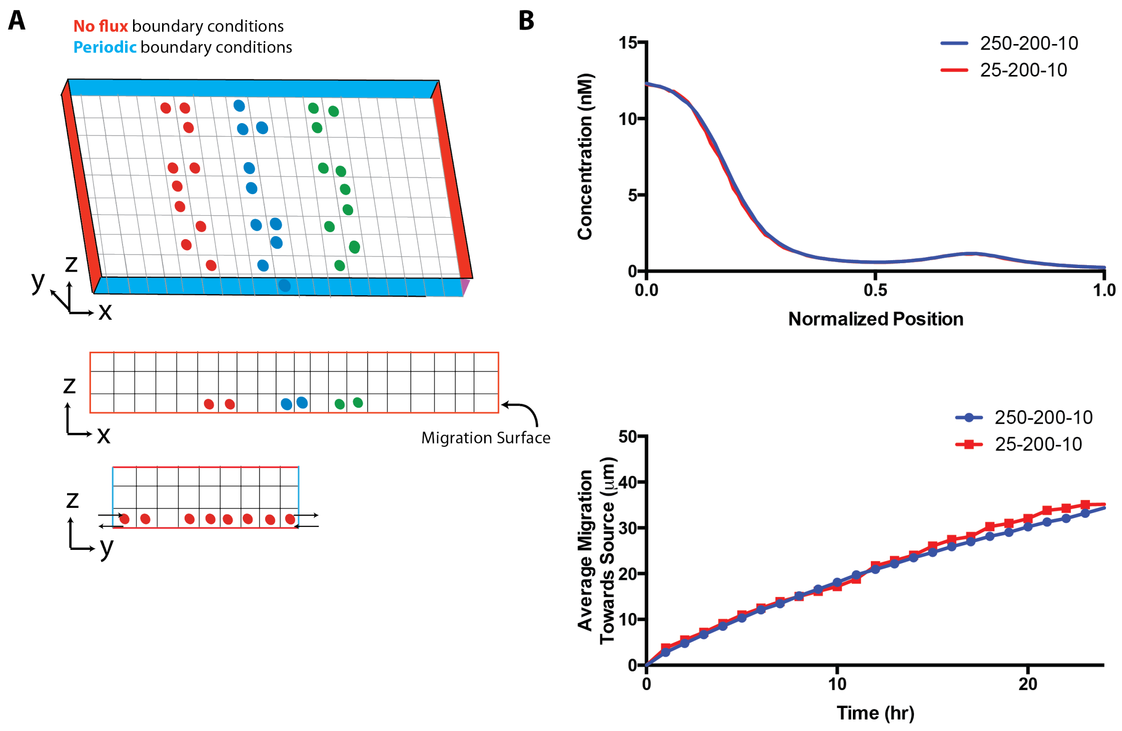

(A) Boundary conditions for agent movement and chemokine diffusion when modeling the source-sink device. (B) Reducing the grid to 25x200x10 gridspaces gives the same gradient calculations and average migration as a grid 10 times as large. Average is representative of 30 simulations.

Figure C in S1 File. Model workflow

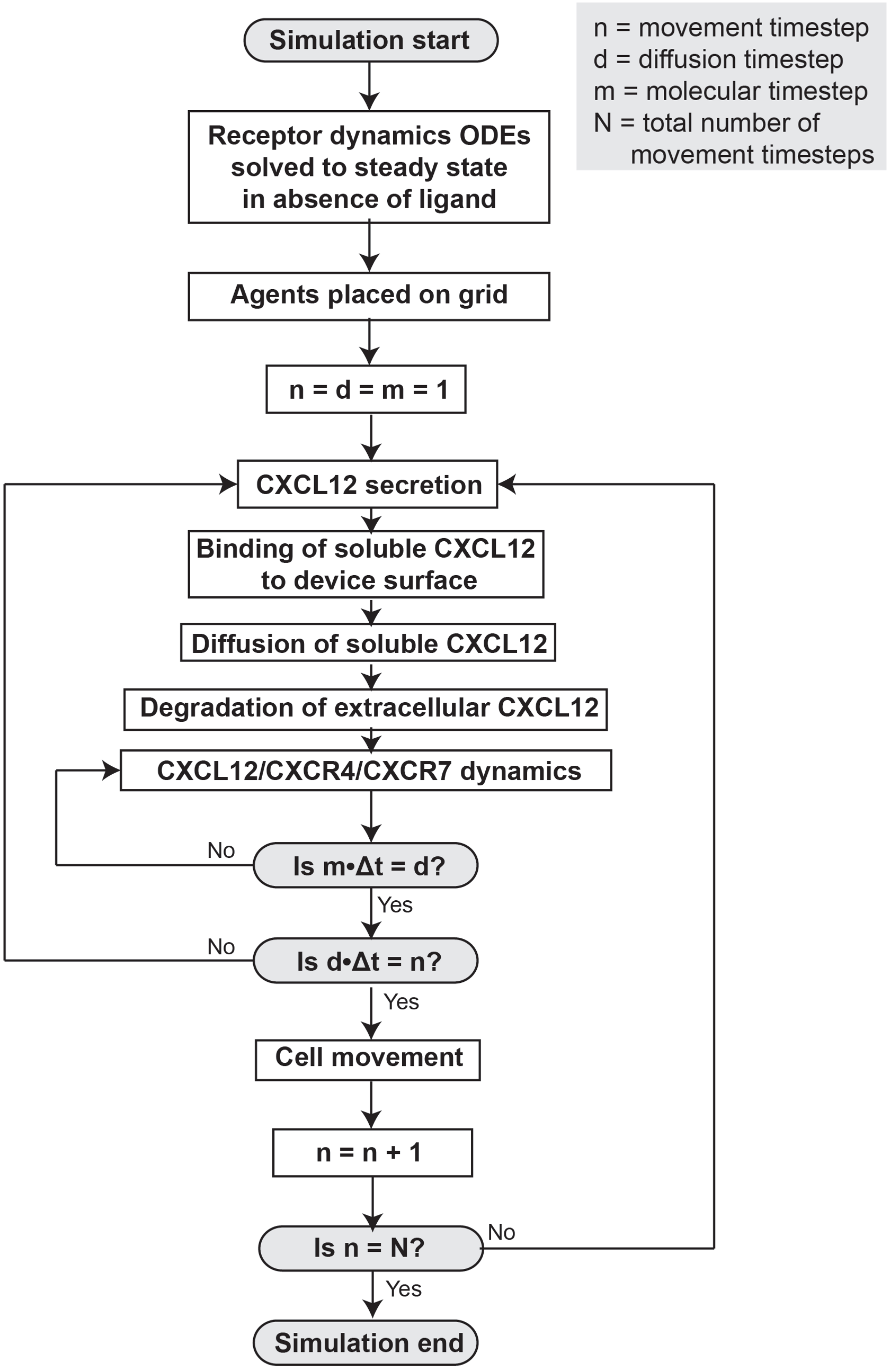

Figure D in S1 File. Validation of movement time step

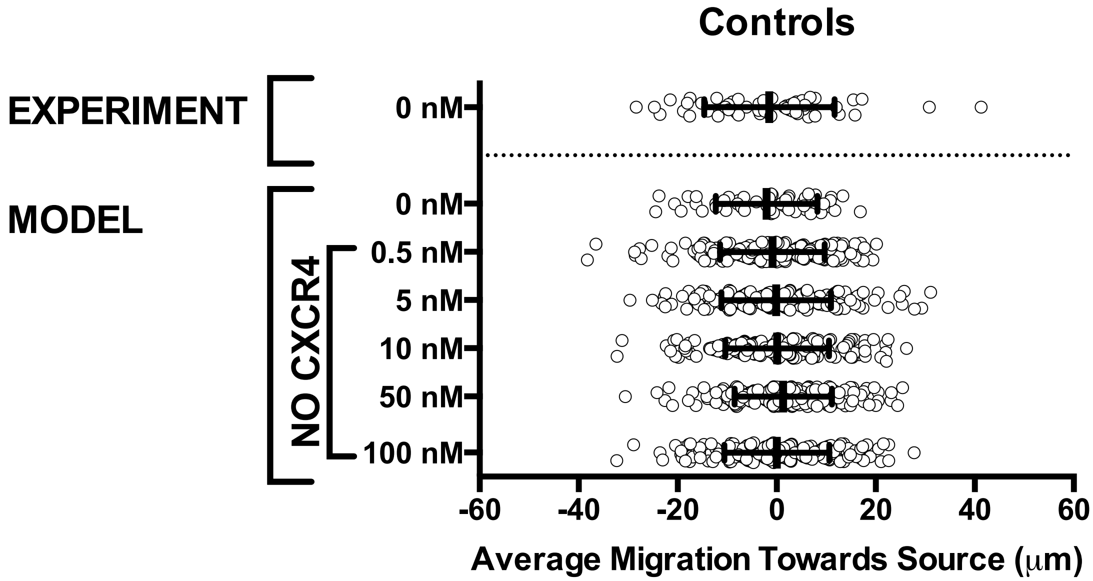

Experimental data reproduced from [1] (Figure 1, E-G at 0% secretion). Each data point (n=54) represents the average position of CXCR4 cells per view field of the device at 24 hours.

CXCR4+ cells in the absence of CXCL12 and cells that do not bear CXCR4 should move randomly (average migration = 0 μm) with a standard deviation that matches random movement within the experimental source-sink device. The model uses the same parameters as in Table A in S1 File, but with (1) an imposed linear CXCL12 gradient with a concentration on the left of the device as indicated in the y-axis, and a concentration of 0 nM on the right of the device and (2) CXCL12+ source cells and CXCR7+ sink cells replaced with cells that did not express the ligand and protein. For the simulation that lacks CXCL12, total number of CXCR4 on CXCR4+ cells is as listed in Table A in S1 File. For all other simulations, total CXCR4 was set to 0. Each data point (n=200) represents the average migration for one device simulation.

In absence of CXCL12 or CXCR4, CXCR4 cells have average migration of 0. Standard deviation is same as experimental.

Figure E in S1 File. Contribution of gradient-shaping events

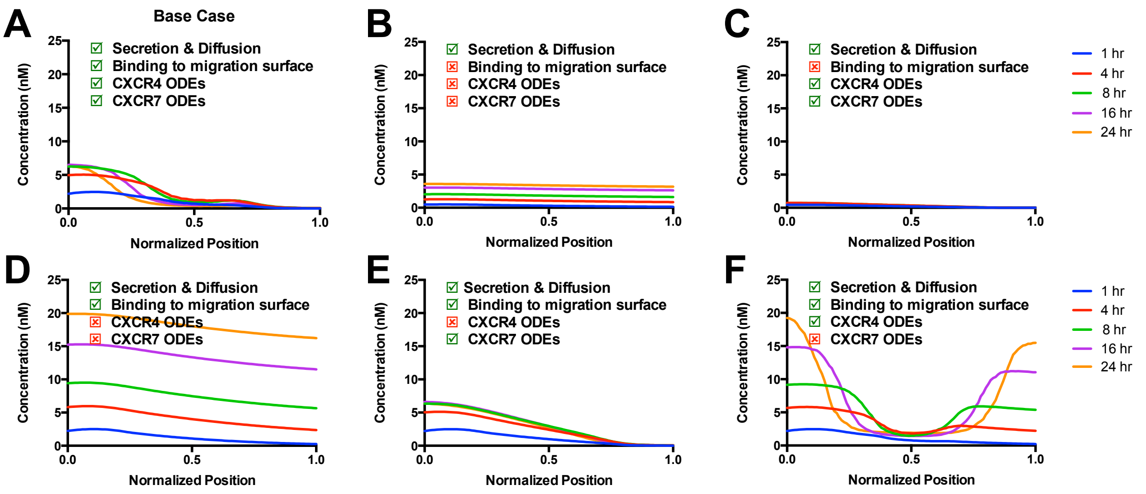

To understand the contribution of cellular and environmental effects on gradient shape over time and position, we systematically "turned off" events from the model. All parameters are as listed in Table A in S1 File. To turn off binding to the migration surface, we set the number of binding sites to 0. To turn off receptor ODEs, we inhibited the call of the ODE functions. Note that secretion and diffusion alone result in relatively shallow, linear gradients that increase in concentration over time. Removing binding to the migration surface limits total CXCL12 concentration. Removing CXCL12-CXCR4 ODEs results in an increase in the gradient in the location of the CXCR4+ cells. Removing CXCL12-CXCR7 ODEs results in an overall increase in CXCL12 concentration across the grid, as well as a significant increase in concentration in the region of the CXCR7+ cells.

Figure F in S1 File. Comparison of gradients within device between CXCL12-α, -β, and -γ

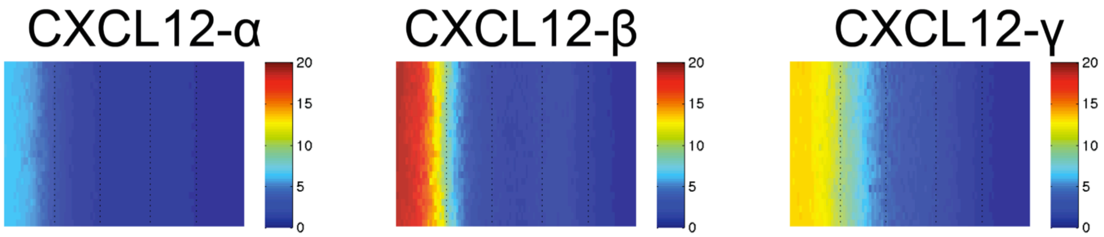

Gradients using parameters in Table 1 and Table A in S1 File. Colorbars indicate total CXCL12 concentration in nM. CXCL12-β has highest overall concentration and steepest gradients compared to CXCL12-α and -γ.

Figure G in S1 File. Effect of CXCR7 on CXCR4 surface receptors

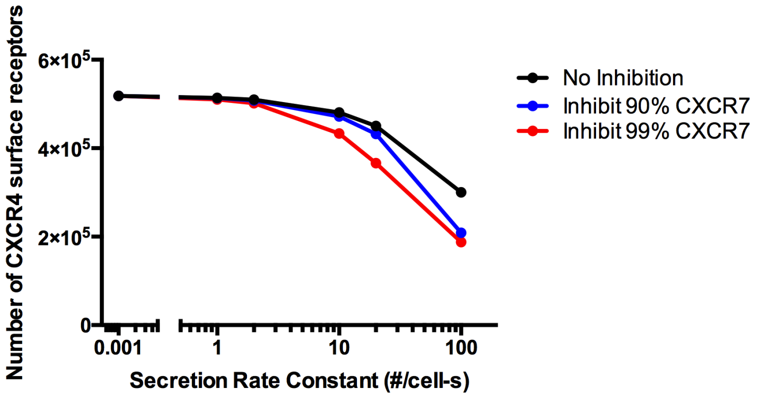

The average number of CXCR4 surface receptors per CXCR4+ cell across a range of CXCL12 secretion rate constants under conditions of no inhibition (all parameters same as listed in Table A in S1 File), inhibition of 90% of CXCR7 receptors, and inhibition of 99% of CXCR7 receptors. Reducing the number of CXCR7 receptors results in a decrease of CXCR4 surface receptors at higher CXCL12 secretion rates, but does not affect CXCR4 surface receptor expression at lower secretion rates. Model data are expressed as mean of 30 replications +/- standard deviation. The maximum standard deviation is ~1000 receptors, thus, error bars are not visible on the plot.

Figure H in S1 File. CXCR4+ movement in gradients of CXCL12-α tumor simulations with adjusted parameters

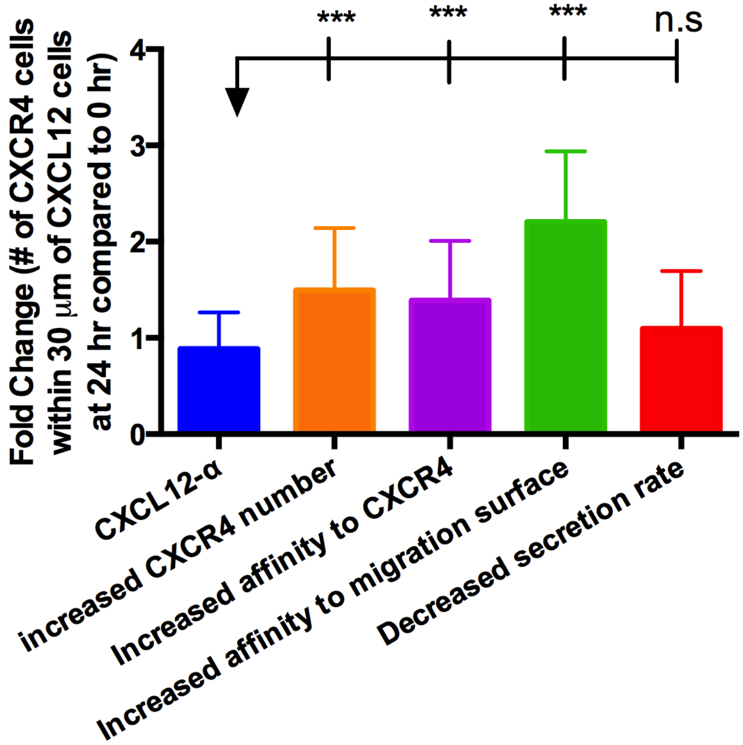

To determine whether CXCL12-α-like parameters can result in CXCR4+ migration in tumor simulations, we increased total CXCR4 from $5x10^3$ to $5x10^4$ receptors/cell, which significantly enhanced CXCR4+ migration. To examine which ligand-related parameters have the largest effect on CXCR4+ migration in tumor simulations, we systemically swapped CXCL12-α parameter values with CXCL12-γ parameter values (increased affinity to CXCR4: $K_{D,R4,L12}$ = 10 nM; increased affinity to migration surface: $K_{D,L,S}$ = 5nM; decreased secretion rate constant: *CXCL12sec* = 5 molecules/cell-s). Both the increase in affinity to the migration surface and to CXCR4 significantly enhance migration. All other parameters and initial conditions were the same as those used in Figure H-J. *** P < 0.0005.

Figure I in S1 File. Tumor simulations using an extended grid

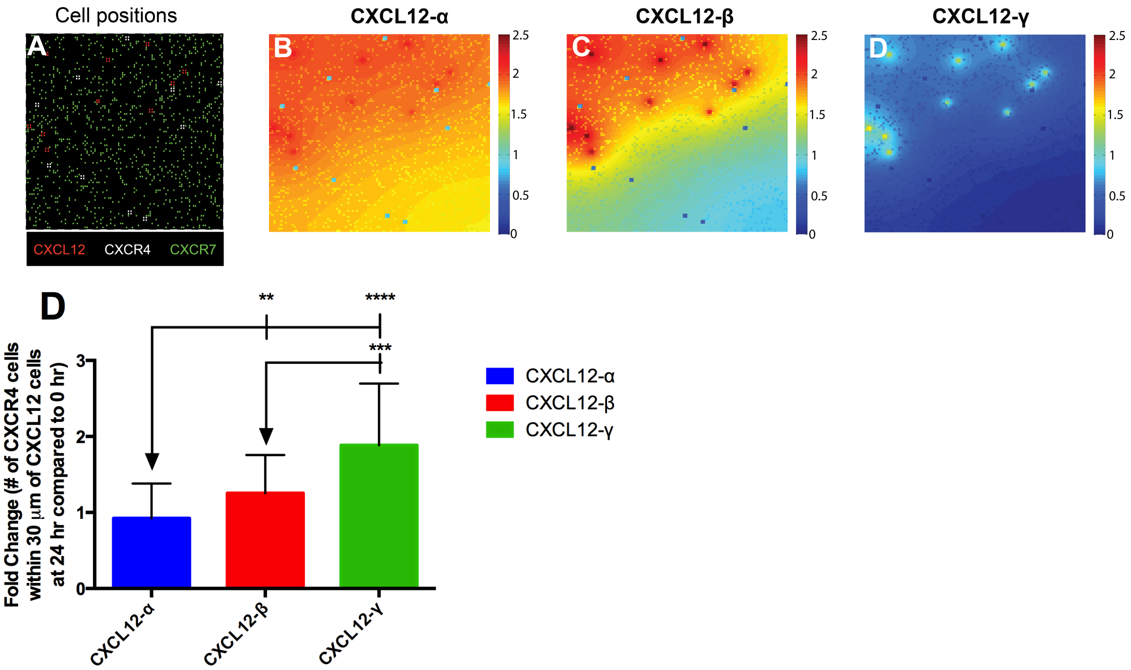

Simulations with the same parameters and initial conditions as Figure 5H-J (5×10^3 total receptors/cell for both CXCR4+ and CXCR7+ cells), but with an extended grid of 100x100x5. (A) Cell positions. Gradients of (B) CXCL12-α, (C) CXCL12-β, (D) CXCL12-γ on the migration surface at 24 hours. Colorbars indicate the total CXCL12 concentration in nM. Similarly to the 2D grid, gradients of CXCL12-γ are characterized by steeper gradients and shorter maintained distances than those of CXCL12-α. (D) Consistent with the results in the 2D grid, CXCR4+ cells move significantly more towards CXCL12+ cells in gradients of CXCL12-γ than CXCL12-β or CXCL12-α. * $P < 0.05$; ** $P < 0.005$; *** $P < 0.0005$.

Table A in S1 File. Model parameters

Table A1 in S1 File. Multi-scale model parameters

Parameter	Description	Value	Literature Range	Reference
Cell density (cells/mm^2)	Density of cells patterned in microfluidic device	2500 (125 cells of each type)		[1]
CXCL12sec (molecules/cell-s)	Secretion rate of CXCL12+ cells (Also shown in Table 1)	20*	15-25	[1]
D_{CXCL12} (cm^2/s)	CXCL12 diffusivity coefficient	$1.5x10^{-6}$	$1.5x10^{-6} – 1.7x10^{-6}$	[2,3]
k_{deg} (s^{-1})	Extracellular CXCL12 degradation rate	$2x10^{-5}$	$6x10^{-5} – 4x10^{-3}$	[4-6]
Total number of surface sites (S+ $L \cdot S$) (#/gridspace)	Number of surface bound sites per gridspace	$5x10^5$	$2.3x10^5$-$1.2x10^6$ #	[7,8]
$K_{D,L,S}$ (nM)	Dissociation constant of CXCL12-α to surface site	100*	93	[9]
$k_{f,L,S}$ (s^{-1})	Forward rate constant of ligand to surface site	0.001^	0.001	[9]
Total CXCR4 (molecules/cell)	Total number of CXCR4 receptors per CXCR4+ cell	$7.1x10^5$		This work
Total CXCR7 (molecules/cell)	Total number of CXCR7 receptors per CXCR7+ cell	$2x10^6$		This work
Total β-arrestin in CXCR4+ cells (molecules/cell)	Total number of β-arrestin per CXCR4+ cell	$5x10^5$		[10]
Total β-arrestin in CXCR7+ cells (molecules/cell)	Total number of β-arrestin per CXCR7+ cell	$7x10^5$&		[10]

* Fit to experimental data, as shown in Figure 3

# Used number of cell surface heparan sulfate sites as a starting point. Sensitivity analysis (using LHS and PRCC as described in Methods) showed no dependence of average CXCR4+ at 24 hr on number of surface sites within this range.

^ Assumed the forward rate constant for ligand binding to a surface site is the same as that for binding to heparan sulfate.

& Increased to reflect that the majority of cell surface receptors on CXCR7+ cells are intracellular.

Table A2 in S1 File. Molecular species involved in ligand-binding and internalization dynamics of CXCR4 and CXCR7

**CXCR4 Species**	**Description**	**CXCR7 Species**	**Description**
R_4 (#/cell)	Free cell-surface CXCR4	R_7 (#/cell)	Free cell-surface CXCR7
L_{12} (nM)	Free extracellular CXCL12	L_{12} (nM)	Free extracellular CXCL12
B_e (#/cell)	Free endogenous β-arrestin 2	B_e (#/cell)	Free endogenous β-arrestin 2
R_{4Be} (#/cell)	R_4 bound to B_e	R_{7Be} (#/cell)	R_7 bound to B_e
C_4 (#/cell)	R_4 bound to L_{12}	C_7 (#/cell)	R_7 bound to L_{12}
C_{4Be} (#/cell)	R_{4Be} bound to L_{12}	C_{7Be} (#/cell)	R_{7Be} bound to L_{12}
R_{4Bei} (#/cell)	Intracellular R_{4Be}	R_{7Bei} (#/cell)	Intracellular R_{7Be}
C_{4Bei} (#/cell)	Intracellular C_{4Be}	C_{7Bei} (#/cell)	Intracellular C_{7Be}
C_{4Beii} (#/cell)	C_{4Bei} after B_e dissociation	R_{7Beii} (#/cell)	R_{7Bei} after B_e dissociation
C_{4Bpii} (#/cell)	C_{4Bpi} after B_p dissociation	C_{7Beii} (#/cell)	C_{7Bei} after trafficking to late endosomes
L_{12i} (#/cell)	Intracellular L_{12}	C_{7Bpii} (#/cell)	C_{7Bpi} after trafficking to late endosomes
		L_{12i} (#/cell)	Intracellular L_{12}

We use the same equation framework and parameters as [10]; however, we do not include any probe-labeled β-arrestin species.

Table A3 in S1 File. Description and values of CXCR4 kinetic and equilibrium parameters

Parameter	Description	Value#	Reported Range^
$k_{f,L12,4}$ ($nM^{-1}s^{-1}$)	Forward rate constant of L_{12} binding R_4/R_{4Be}	2.1 $x10^{-3}$	2.8 – 6.7 $x10^{-3}$
$k_{f,B,4}$ ($(\#/cell)^{-1}s^{-1}$)	Forward rate constant of B_e binding R_4/C_4	8.5 $x10^{-9}$	10^{-8} – 10^{-6}
$K_{D,R4,L12}$ (nM)	Equilibrium dissociation constant of L_{12} binding R_4	40	2-27
$K_{D,R4,B}$ (#/cell)	Equilibrium dissociation constant of B_e from R_4	7.8 $x10^6$	10^4 – 10^6
$K_{D,C4,B}$ (#/cell)	Equilibrium dissociation constant of B_e from C_4	5.1 $x10^6$	10^4 – 10^6
$k_{e,R4B}$ (s^{-1})	R_{4Be} internalization rate constant	2.3 $x10^{-3}$	1 – 2 $x10^{-3}$
$k_{e,C4B}$ (s^{-1})	C_{4Be} internalization rate constant	4.7 $x10^{-3}$	3 $x10^{-3}$
$k_{off,B,4}$ (s^{-1})	Dissociation rate constant of B_e from C_{4Bei}	7.4 $x10^{-4}$	
$k_{rec,R4Bi}$ (s^{-1})	R_{4Bei} recycling rate constant	$3x10^{-4}$ *	10^{-4} – 10^{-3}
$k_{deg,C4Bii}$ (s^{-1})	C_{4Beii} degradation rate constant	1.0 $x10^{-4}$	10^{-5} – 10^{-4}
$k_{deg,L12i}$ (s^{-1})	L_{12i} degradation rate constant	1.0 $x10^{-4}$	10^{-4} – 10^{-3}

All CXCR4 parameter values are taken from [10] unless otherwise noted.
^ This reported range that was examined in [10] is the same used for sensitivity analysis. When the parameter value is outside the literature range (which happens because it was fit to experimental data), we extend the sensitivity analysis range to that value.
* This value was increased from $6.9x10^{-5}$ (value in [10]) to $3x10^{-4}$ (4x increase) to better capture the slow decrease in surface receptor numbers seen in long time frame experiments.

Table A4 in S1 File. Description and values of CXCR7 kinetic and equilibrium parameters.

Parameter	Description	Value#	Reported Range^
$k_{f,L12,7}$ ($nM^{-1}s^{-1}$)	Forward rate constant of L_{12} binding R_7/R_{7Be}	1.4 x10^{-3}	2.8 – 6.7 x10^{-3}
$k_{f,B,7}$ ($(\#/cell)^{-1}s^{-1}$)	Forward rate constant of B_e binding R_7/C_7	1.4 x10^{-8}	10^{-8} – 10^{-6}
$K_{D,R7,L12}$ (nM)	Equilibrium dissociation constant of L_{12} binding R_7	0.84	0.2 – 0.4
$K_{D,R7,B}$ (#/cell)	Equilibrium dissociation constant of B_e from R_7	2.3 x10^{6}	10^{4} – 10^{6}
$K_{D,C7,B}$ (#/cell)	Equilibrium dissociation constant of B_e from C_7	6.5 x10^{5}	10^{4} – 10^{6}
$k_{e,R7B}$ (s^{-1})	R_{7Be} internalization rate constant	3.9 x10^{-3}	1 – 2 x10^{-3}
$k_{e,C7B}$ (s^{-1})	C_{7Be} internalization rate constant	2.1 x10^{-3}	3 x10^{-3}
$k_{off,B,7}$ (s^{-1})	Dissociation rate constant of B_e from R_{7Bei}	2.5 x10^{-3}	
$k_{e,C7Bi}$ (s^{-1})	Rate constant of trafficking of C_{7Bei} to late endosomes	5.5 x10^{-4}	
$k_{rec,R7Bii}$ (s^{-1})	R_{7Beii} recycling rate constant	1.1 x10^{-3}	10^{-4} – 10^{-3}
$k_{rec,C7Bii}$ (s^{-1})	C_{7Beii} recycling rate constant	2.8 x10^{-4}	10^{-4} – 10^{-3}
$k_{deg,L12i}$ (s^{-1})	L_{12i} degradation rate constant	1.0 x10^{-4}	10^{-4} – 10^{-3}

All CXCR7 parameter values are taken from [10].
^ This reported range that was examined in [10] is the same used for sensitivity analysis. When the parameter value is outside the literature range (which happens because it was fit to experimental data), we extend the sensitivity analysis range to that value.

Table A5 in S1 File. Ordinary differential equations describing events of ligand binding and receptor dynamics

Cellular Event	CXCR4+ cells	CXCR7+ cells
Ligand binding to free receptors	$v_1 = k_{f,L_{12},4}([R_4][L_{12}] - K_{D,R_4,L_{12}}[C_4])$	$v_2 = k_{f,L_{12},7}([R_7][L_{12}] - K_{D,R_7,L_{12}}[C_7])$
Ligand binding to receptor-β-arrestin complexes	$v_3 = k_{f,L_{12},4}([R_{4B_e}][L_{12}] - K_{D,R_{4B},L_{12}}[C_{4B_e}])$	$v_4 = k_{f,L_{12},7}([R_{7B_e}][L_{12}] - K_{D,R_{7B},L_{12}}[C_{7B_e}])$
β-arrestin binding to free receptors	$v_7 = k_{f,B,4}([R_4][B_e] - K_{D,R_4,B}[R_{4B_e}])$	$v_8 = k_{f,B,7}([R_7][B_e] - K_{D,R_7,B}[R_{7B_e}])$
β-arrestin binding to ligand-bound receptors	$v_{11} = k_{f,B,4}([C_4][B_e] - K_{D,C_4,B}[C_{4B_e}])$	$v_{12} = k_{f,B,7}([C_7][B_e] - K_{D,C_7,B}[C_{7B_e}])$
Internalization of cell surface receptor-β-arrestin complexes	$v_{15} = k_{e,R_{4B}}[R_{4B_e}]$	$v_{16} = k_{e,R_{7B}}[R_{7B_e}]$
	$v_{19} = k_{e,C_{4B}}[C_{4B_e}]$	$v_{20} = k_{e,C_{7B}}[C_{7B_e}]$
Dissociation of β-arrestin from internalized receptor-β-arrestin complexes	$v_{23} = k_{off,B,7}[R_{7B_ei}]$	$v_{25} = k_{off,B,4}[C_{4B_ei}]$
Trafficking of internalized receptor-β-arrestin complexes to late endosomes	N/A	$v_{27} = k_{e,C_{7Bi}}[C_{7B_ei}]$
Recycling of internalized receptors	$v_{29} = k_{rec,R_{4Bi}}[R_{4B_ei}]$	$v_{30} = k_{rec,R_{7Bii}}[R_{7B_eii}]$
	N/A	$v_{33} = k_{rec,C_{7Bii}}[C_{7B_eii}]$
Degradation of internalized receptors and ligand	$v_{35} = k_{deg,C_{4Bii}}[C_{4B_eii}]$	
Degradation of L_{12i}	$v_{37} = k_{deg,L_{12i}}[L_{12i}]$	$v_{37} = k_{deg,L_{12i}}[L_{12i}]$

Numbering of equations is consistent with that used in [10].

Table A6 in S1 File. Ordinary Differential Equations describing the change in the concentration of species in CXCR4+ and CXCR7+ cells over time

CXCR4 Equations (#/cell/s)	CXCR7 Equations (#/cell/s)
$\frac{d[R_4]}{dt} = -v_1 - v_7 - v_9 + v_{29} + v_{31}$	$\frac{d[R_7]}{dt} = -v_2 - v_8 - v_{10} + v_{30} + v_{32} + v_{33} + v_{34}$
$\frac{d[R_{4B_e}]}{dt} = +v_7 - v_3 - v_{15}$	$\frac{d[R_{7B_e}]}{dt} = +v_8 - v_4 - v_{16}$
$\frac{d[C_4]}{dt} = +v_1 - v_{11} - v_{13}$	$\frac{d[C_7]}{dt} = +v_2 - v_{12} - v_{14}$
$\frac{d[C_{4B_e}]}{dt} = +v_3 + v_{11} - v_{19}$	$\frac{d[C_{7B_e}]}{dt} = +v_4 + v_{12} - v_{20}$
$\frac{d[R_{4B_ei}]}{dt} = +v_{15} - v_{29}$	$\frac{d[R_{7B_ei}]}{dt} = +v_{16} - v_{23}$
$\frac{d[C_{4B_ei}]}{dt} = +v_{19} - v_{25}$	$\frac{d[C_{7B_ei}]}{dt} = +v_{20} - v_{27}$
$\frac{d[C_{4B_eii}]}{dt} = +v_{25} - v_{35}$	$\frac{d[C_{7B_eii}]}{dt} = +v_{27} - v_{33}$
$*\frac{d[L_{12}]}{dt} = (-v_1 - v_3 - v_5)\times\frac{n_4\times10^9}{V\times N_{Av}}$	$*\frac{d[L_{12}]}{dt} = (-v_2 - v_4 - v_6)\times\frac{n_7\times10^9}{V\times N_{Av}}$
$\frac{d[L_{12i}]}{dt} = +v_{19} + v_{21} - v_{37}$	$\frac{d[L_{12i}]}{dt} = +v_{20} + v_{22} - v_{37}$
$\frac{d[B_e]}{dt} = -v_7 - v_{11} + v_{15} + v_{25}$	$\frac{d[B_e]}{dt} = -v_8 - v_{12} + v_{23} + v_{33}$
	$\frac{d[R_{7B_eii}]}{dt} = +v_{23} - v_{30}$

*Units for this equation are (nM/s)

N_{AV}:Avogadro's number

Table B in S1 File. Total receptor numbers for the MDA-MB-231 cells transfected to express CXCR4 and CXCR7 used in the device as determined by flow cytometry.

Cell Type	Total receptor number (#/cell)
**CXCR4+**	$7.1 x 10^5 \pm 1.0 x 10^5$
**CXCR7+**	$5.8 x 10^6 \pm 6.5 x 10^5$

Table C in S1 File. Sensitivity Analysis for CXCR4 and CXCR7 molecular parameters

	**CXCR4 Average Migration**	**Average Concentration**	**Surface CXCR4**	**Total CXCR4**	**Surface CXCR7**
**Ligand binding**					
$K_{D,R4,L12}$	---	---	+++		
$K_{D,R7,L12}$		+++	---	---	
**β-arrestin binding**					
$K_{D,R4,B}$	+++	+++		--	
$K_{D,C4,B}$	++	---	+++	++	
$K_{D,R7,B}$	+++	---	+++	+++	+++
$K_{D,C7,B}$	---	+++	---	---	
**Internalization**					
$k_{e,R4B}$	---		--		
$k_{e,C4B}$		+++		+++	+
$k_{e,R7B}$		+++	---	---	---
$k_{e,C7B}$		---	+++	+++	
**Recycling and Degradation**					
$k_{rec,R4Bi}$	+++		+++		
$k_{deg,C4Bii}$					
$k_{off,B,4}$	---	+	---	---	--
$k_{rec,R7Bii}$	++	---	+++	+++	+++
$k_{rec,C7Bii}$					
$k_{offB,7}$		-	++	++	--

Each parameter was varied within the range reported in Table A4 in S1 File. Sign represents a positive or negative PRCC value. The number of signs represents the significance of the correlation:

+/- : $10^{-5} < p < 10^{-2}$

++/-- : $10^{-12} < p < 10^{-5}$

+++/--- : $p < 10^{-12}$

S1 File References

1. Cavnar SP, Ray P, Moudgil P, Chang SL, Luker KE, et al. (2014) Microfluidic source-sink model reveals effects of biophysically distinct CXCL12 isoforms in breast cancer chemotaxis. Integr Biol (Camb) 6: 564-576.
2. Torisawa YS, Mosadegh B, Bersano-Begey T, Steele JM, Luker KE, et al. (2010) Microfluidic platform for chemotaxis in gradients formed by CXCL12 source-sink cells. Integr Biol (Camb) 2: 680-686.
3. Lin F, Butcher EC (2006) T cell chemotaxis in a simple microfluidic device. Lab Chip 6: 1462-1469.
4. Bellmann-Sickert K, Beck-Sickinger AG (2011) Palmitoylated SDF1alpha shows increased resistance against proteolytic degradation in liver homogenates. ChemMedChem 6: 193-200.
5. Lambeir AM, Proost P, Durinx C, Bal G, Senten K, et al. (2001) Kinetic investigation of chemokine truncation by CD26/dipeptidyl peptidase IV reveals a striking selectivity within the chemokine family. J Biol Chem 276: 29839-29845.
6. Ray JC, Flynn JL, Kirschner DE (2009) Synergy between individual TNF-dependent functions determines granuloma performance for controlling Mycobacterium tuberculosis infection. J Immunol 182: 3706-3717.
7. Incardona F, Calvo F, Fauvel-Lafeve F, Legrand Y, Legrand C (1993) Involvement of thrombospondin in the adherence of human breast-adenocarcinoma cells: a possible role in the metastatic process. Int J Cancer 55: 471-477.
8. Jacobs AL, Julian J, Sahin AA, Carson DD (1997) Heparin/heparan sulfate interacting protein expression and functions in human breast cancer cells and normal breast epithelia. Cancer Res 57: 5148-5154.
9. Laguri C, Sadir R, Rueda P, Baleux F, Gans P, et al. (2007) The novel CXCL12gamma isoform encodes an unstructured cationic domain which regulates bioactivity and interaction with both glycosaminoglycans and CXCR4. PLoS One 2: e1110.
10. Coggins NL, Trakimas D, Chang SL, Ehrlich A, Ray P, et al. (2014) CXCR7 Controls Competition for Recruitment of beta-Arrestin 2 in Cells Expressing Both CXCR4 and CXCR7. PLoS One 9: e98328.
